# The paradox of HBV evolution as revealed from a 16^th^ century mummy

**DOI:** 10.1371/journal.ppat.1006750

**Published:** 2018-01-04

**Authors:** Zoe Patterson Ross, Jennifer Klunk, Gino Fornaciari, Valentina Giuffra, Sebastian Duchêne, Ana T. Duggan, Debi Poinar, Mark W. Douglas, John-Sebastian Eden, Edward C. Holmes, Hendrik N. Poinar

**Affiliations:** 1 Marie Bashir Institute for Infectious Diseases and Biosecurity, Charles Perkins Centre, School of Life and Environmental Sciences and Sydney Medical School, The University of Sydney, Sydney, New South Wales, Australia; 2 McMaster Ancient DNA Centre, Department of Anthropology, McMaster University, Hamilton, ON, Canada; 3 Division of Paleopathology, Department of Translational Research on New Technologies in Medicine and Surgery, University of Pisa, Pisa, Italy; 4 Department of Biochemistry and Molecular Biology, Bio21 Molecular Science and Biotechnology Institute, University of Melbourne, Parkville, Victoria, Australia; 5 Storr Liver Centre, The Westmead Institute for Medical Research, The University of Sydney and Westmead Hospital, Westmead, New South Wales, Australia; 6 Michael G. DeGroote Institute for Infectious Disease Research and the Department of Biochemistry, McMaster University, Hamilton, ON, Canada; 7 Humans and the Microbiome Program, Canadian Institute for Advanced Research, Toronto, ON, Canada; Rutgers University, UNITED STATES

## Abstract

Hepatitis B virus (HBV) is a ubiquitous viral pathogen associated with large-scale morbidity and mortality in humans. However, there is considerable uncertainty over the time-scale of its origin and evolution. Initial shotgun data from a mid-16^th^ century Italian child mummy, that was previously paleopathologically identified as having been infected with Variola virus (VARV, the agent of smallpox), showed no DNA reads for VARV yet did for hepatitis B virus (HBV). Previously, electron microscopy provided evidence for the presence of VARV in this sample, although similar analyses conducted here did not reveal any VARV particles. We attempted to enrich and sequence for both VARV and HBV DNA. Although we did not recover any reads identified as VARV, we were successful in reconstructing an HBV genome at 163.8X coverage. Strikingly, both the HBV sequence and that of the associated host mitochondrial DNA displayed a nearly identical cytosine deamination pattern near the termini of DNA fragments, characteristic of an ancient origin. In contrast, phylogenetic analyses revealed a close relationship between the putative ancient virus and contemporary HBV strains (of genotype D), at first suggesting contamination. In addressing this paradox we demonstrate that HBV evolution is characterized by a marked lack of temporal structure. This confounds attempts to use molecular clock-based methods to date the origin of this virus over the time-frame sampled so far, and means that phylogenetic measures alone cannot yet be used to determine HBV sequence authenticity. If genuine, this phylogenetic pattern indicates that the genotypes of HBV diversified long before the 16^th^ century, and enables comparison of potential pathogenic similarities between modern and ancient HBV. These results have important implications for our understanding of the emergence and evolution of this common viral pathogen.

## Author summary

Hepatitis B virus (HBV) exerts formidable morbidity and mortality in humans. We used ancient DNA techniques to recover the complete genome sequence of an HBV from the mummified remains of a child discovered in the 16^th^ century from Naples, Italy. Strikingly, our analysis of this specimen resulted in two contrasting findings: while the damage patterns lend credence to this HBV sequence being authentically 16^th^ century, phylogenetic analysis revealed a close relationship to recently sampled viruses as expected if the sequence were a modern contaminant. We reconcile these two observations by showing that HBV evolution over the last ~450 years is characterized by a marked lack of temporal structure that hinders attempts to resolve the evolutionary time-scale of this important human pathogen.

## Introduction

The comparative analysis of viral genomes provides a wide and informative view of evolutionary patterns and processes. In particular, viruses often evolve with sufficient rapidity to inform on evolutionary processes over the timespan of direct human observation (weeks and days) [1–3]. Importantly, recent technical developments in next generation sequencing [4, 5] and ancient DNA (aDNA) recovery [6] have enabled the rigorous study of nucleotide sequences from increasingly older historical, archaeological and paleontological samples. Consequently, aDNA sequences can now facilitate the study of more slowly evolving pathogen populations, from which recent samples display limited sequence diversity, by permitting an expansion of the indirectly observable timespan to that of centuries [7, 8]. Hence, viral and bacterial genomes recovered from such ancient samples have the potential to reveal the etiological agents associated with past pandemics, as well as important aspects of the long-term patterns and processes of evolutionary change within pathogen populations.

To date, investigations of ‘ancient’ viruses have been limited in number and scope. Those focusing on pathogens sampled prior to 1900 have considered four human viruses: variola virus (VARV, the agent of smallpox) [8, 9], human papillomavirus [10], human T-cell lymphotropic virus [11], and hepatitis B virus (HBV) [12, 13]. Similarly, aDNA techniques have been used in studies of major 20^th^ century epidemics of influenza virus and human immunodeficiency virus [14, 15]. Taken together, these studies have helped to clarify the causative agents of specific outbreaks, whether ancient strains differ markedly from recent ones, and the evolutionary and epidemiological processes that have likely shaped virus diversity. Moreover, they have provided key information on the dynamics of evolutionary change, including the ‘time-dependent’ nature of viral evolution in which estimates of evolutionary rates are routinely elevated toward the present and decline toward the past due to a combination of unpurged transient deleterious mutations in the short-term and site saturation in the long-term [16].

A major challenge for any study of aDNA is the accumulation of post-mortem damage in the genome of interest. This damage includes fragmentation, nucleotide deamination, and polymerase-blocking lesions, such as molecular cross-linking, resulting from enzymatic and chemical reactions [17–19]. Certain environmental conditions, including desiccation of organic material and low ambient temperatures, can inhibit the activity of some endonucleases and environmental microorganisms, although oxidative and hydrolytic processes will continue to occur in all conditions at variable rates depending on the preservational context [20]. However, the predictably recurring forms of damage, such as the tendency for cytosine deamination to occur more often near the 3’ and 5’ ends of fragmented DNA molecules, also provide a means of addressing questions of contamination and inferring the authenticity of a recovered sequence through statistical pattern analysis [21].

For rapidly evolving genomes, such as those from viruses, phylogenetic analysis can provide another means of establishing provenance. In particular, as the rate of evolutionary change is high in many viruses [22], “ancient” viruses should generally fall closer to the root of a tree than their modern relatives. However, a complicating factor is that any phylogenetic inference that aims to determine evolutionary rates or time-scale is only meaningful when the virus in question exhibits clear temporal structure in the phylogeny, such that it is evolving in an approximately clock-like manner over the time-scale of sampling. Because DNA viruses generally exhibit lower rates of evolutionary change than RNA viruses [22], such populations tend to require larger sampling time-frames to discern temporal structure and clock-like behavior [16].

Hepatitis B virus (HBV) (family *Hepadnaviridae*) presents a compelling case of the complexities of analyzing the evolution of DNA viruses. Despite considerable effort, the evolutionary rate and time of origin of this important human pathogen remain uncertain, even though it is chronically carried by approximately 350 million people globally, with almost one million people dying each year as a result [23]. Particularly puzzling is that although HBV utilizes an error-prone reverse transcriptase (RT) for replication, estimates of its evolutionary rate are generally low, yet also highly variable. For instance, mean rate estimates of 2.2 × 10^−6^ nucleotide substitutions per site per year (subs/site/year) have been derived from long-term studies utilizing internal node calibrations on phylogenetic trees built using conserved regions of the viral genome [24], while rates of up to 7.72 × 10^−4^ subs/site/year have been recorded within a single patient [25] and pedigree-based studies have returned mean rates of 7.9 × 10^−5^ subs/site/year [26]. As the highest evolutionary rates are observed in the short-term, this pattern is consistent with both relatively high background mutation rates and a time-dependent pattern of virus evolution in which rates are elevated toward the present due to incomplete purifying selection [16].

HBV is also genetically diverse, comprising ten different genotypes designated A–J, as well as additional subgenotypes within genotypes A–D and F [27]. Intra-genotypic sequence differences average 8%, while subgenotypes differ by an average of 4% [28, 29]. The HBV genotypes differ in their geographic distributions. Genotype A is most prevalent in northwestern Europe and the United States, while genotypes B and C predominate in Asia, and genotype D in the Mediterranean basin, including Italy, as well as the Middle East and India. Similarly, genotype E is mostly seen in west Africa, genotype F in South and Central America, genotype G in the USA and France, and genotype H in Mexico and South America [30]. The more recently described genotype I has been identified in Vietnam [31] and Laos [32], while the one example of genotype J was isolated from a Japanese sample [33].

With over half of the nucleotide coding for more than one protein, the physical constraints of the HBV genome are likely to have a major impact on evolutionary dynamics. Partially double-stranded, the relaxed-circular DNA genome averages ~3200 bp in length for the longer strand and ~1700–2800 for the shorter, comprised of four overlapping open reading frames (ORFs). These ORFs encode seven proteins; the pre-core and core proteins, three envelope proteins (small, medium, and large S), the reverse transcriptase (RT, the polymerase), and an X protein that is thought to mediate a variety of virus-host interactions [34, 35]. As noted above, the replication of HBV involves the use of RT, an enzyme that has no associated proofreading mechanism, such that mutational errors are expected to be frequent [25, 36]. However, due to the overlapping ORFs, many mutations are likely to be non-synonymous and therefore purged by purifying selection.

Considerable uncertainty remains as to when HBV entered human populations and when it differentiated into distinct genotypes. Given its global prevalence and the presence of related viruses in other mammals including non-human primates, it is commonly believed that the virus has existed in human populations for many thousands of years [37]. Further, recent studies have shown that the long-term evolutionary history of the *Hepadnaviridae* is shaped by a complex mix of long-term virus-host co-divergence and cross-species transmission [38]. Hence, it seems reasonable to conclude that HBV diversified within geographically isolated human populations following long-term continental migrations [24].

Ancient DNA has the potential to provide a new perspective on the evolutionary history of HBV. Notably, HBV has been sequenced from a Korean mummy radiocarbon dated to 330 years BP (±70 years) which translates to ca.1682 (with an error range of 1612–1752) [12]. Phylogenetic analysis of this sequence (GenBank accession JN315779) placed it within the modern diversity of genotype C, which is common to Asia. This phylogenetic position is compatible with low long-term rates of evolutionary change in HBV such that the virus has existed in human populations for many thousands of years, with genotypes diversifying over this time-scale [24]. However, the authenticity of this sequence, and hence the evolutionary time-scale it infers, remains uncertain, as deamination analysis and read length distribution were not reported as the data were generated using PCR based methodologies. In addition, as genotype C is common in modern Asian populations [37] and the mummy sequence (JN315779) clusters closely with modern sequences, there is no phylogenetic evidence to support the historical authenticity of this sequence.

For aDNA to resolve the origins of HBV it is of paramount importance to determine whether ancient samples can be used to calibrate the molecular clock to provide more accurate estimates of the time-scale of HBV origins and evolution. To this end, we report the detailed study of a complete HBV genome sampled from a 16^th^ century Italian mummy.

## Results

### A 16^th^ century mummy from the Basilica of Saint Domenico Maggiore, Naples, Italy

**Fig 1:**
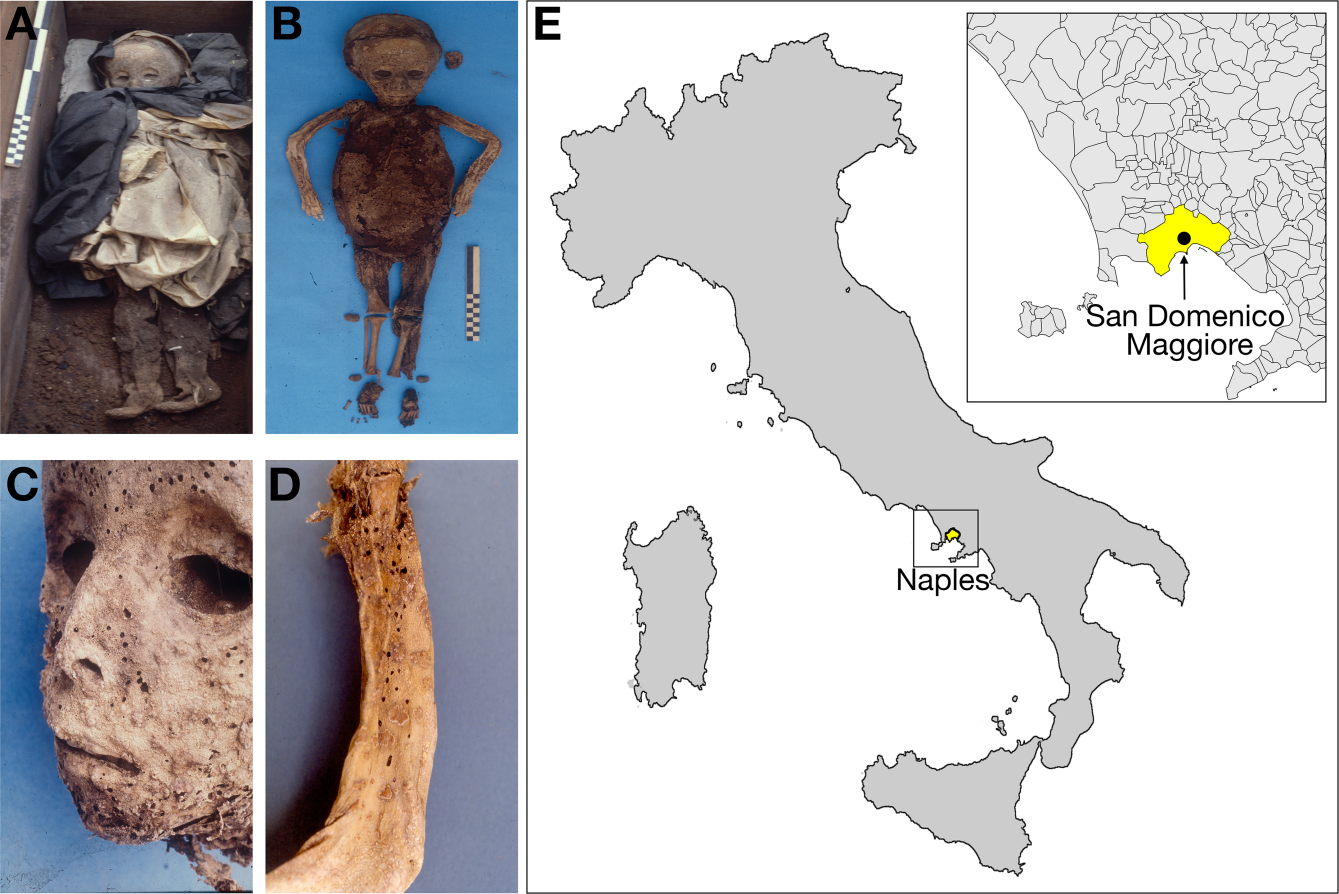
Location and subject. Images of NASD24 showing (A) the mummy wearing funerary dress in the coffin, (B) the child’s mummified body prior to the autopsy, (C) the vesicopustular rash as evident on the facial features, and (D) this rash as evident on the arm. (E) Map displaying location of the Basilica of Saint Domenico Maggiore in Naples, Italy. The map of Italy was constructed in Matlab v2016b using the landareas.shp to define the outline of the landmass of Italy. The shapefile obtained from the Italian National Statistics Institute (ISTAT) (http://www3.istat.it/dati/catalogo/20061102_00/) was used to define the Italian administrative borders as displayed within the inset focused on Naples and showing the exact location of San Domenico Maggiore (latitude = 40.849027, longitude = 14.254418).

We sampled the remains of an unidentified child mummy, approximately two years of age, found in the sacristy of the Basilica of Saint Domenico Maggiore in Naples, Italy, and exhumed between 1983 and 1985 [39] (Fig 1). This mummy is described in previous studies as mummy no. 24 (NASD24) [40]. Radiocarbon (^14^C) dating indicates that this mummy is 439 years old (± 60 years), thereby placing it to 1569 CE ± 60 years [39]. Evidence from the funerary context, including the dress style [41, 42], particularities of the mummification technique, and the known identities and historical records of other mummies, agree with this time frame [39, 40]. Shotgun analysis of a suite of mummified remains from this site showed that one of the remains, those of NASD24, yielded sequence reads mapping closely to viral sequences from the *Hepadnaviridae* (Table 1, S1 Table).

**Table 1:** HBV reads identified in shotgun sequencing results from various NASD24 samples.

Library	Tissue source	Total no. reads (R1 + R2)	No. reads trimmed + merged	No. unique reads* mapped X65257	% X65257 covered at least 1x	Average coverage
LM1	Distal femur	551452	286878	18	21.8	0.3
LM2	Rib with skin	561922	290139	0	0	0
LM3	Frontoparietal bone with skin	2987034	1533717	8	11.2	0.2
LM10	Abdominal skin	3127872	1750882	0	0	0
LM11	Thigh muscle	2957006	1581829	0	0	0
LM12	Thoracic skin	2968202	1545427	3	3.3	0
LM14	Leg skin	1663828	906399	0	0	0
LM15	Scalp	1820452	1009055	0	0	0
P03MMSG	Abdominal skin	1845984	944426	0	0	0
P04MMSG	Scalp	560	413	0	0	0

*‘Unique reads’ refer to those merged or properly paired with a minimum length of 30bp.

Prior to the deposition in a coffin on a suspended passageway of the sacristy, the body of mummy NASD24 was eviscerated and embalmed. Records indicate the mummy was left undisturbed from 1594 [40]. Notably, the autopsy identified a diffuse vesiculopustular rash on the arm, body and face [40]. Paleopathological interpretation of this rash identified it as evidence of a possible smallpox infection. Electron microscopic images produced in an earlier study of pustular tissue homogenates from this mummy showed egg-shaped, dense structures, and positive results in immunostaining with protein-A/gold complex of ultrathin sections of pustular skin incubated with human anti-vaccinia-virus antiserum supported the presence of a poxvirus [43]. However, in our study, we also attempted SEM analysis of the tissue samples of NASD24 and did not find evidence of particles resembling either VARV or HBV in their dimensions, though did discover particles consistent with an unknown viral origin (S1 Fig). We are uncertain how mummification (or later processes involved in preservation and preparation for EM analysis) may have affected the physical appearance of viral particles.

### aDNA extraction, library preparation, enrichment and sequencing

We extracted total DNA from samples of the distal femur, skin attached to a rib, skin attached to the frontoparietal bone, thigh muscle, temporo-maxillary skin, and leg skin of mummy NASD24 (Table 2) using a modified organic phenol-chloroform-isoamyl method [44] in dedicated aDNA facilities at McMaster University, Hamilton, Canada. These extracts were converted into double-stranded (ds) Illumina indexed sequencing libraries both with and without uracil DNA glycosylase (UDG) treatment and enriched for HBV, VARV, and mitochondrial genomes using in-solution bait sets. All libraries were sequenced on an Illumina HiSeq 1500 platform. We generated a total of 1,041,774 reads for the UDG-treated LM01 library (distal femur) and 3,869,248 for the non-UDG-treated LM01 library (Table 2). Of the trimmed and merged reads (minimum 30 base pairs in length), 4,338 unique reads mapped to HBV D3 genotype X65257 from the UDG-treated library and 4,360 from the non-UDG-treated library. A smaller number of reads mapped to X65257 from other samples (Table 2). A total of 9610 reads from the distal femur, the skin attached to the rib and the fronto-parietal bone, the thigh muscle, the temporo-maxillary skin and the leg skin were pooled for further analysis using a consensus sequence (Table 2, S1 Table).

**Table 2:** Total reads and coverage for HBV, mtDNA, and/or VARV genomes from various tissue samples.

Library	Tissue source	Total no. reads (R1 + R2)	No. reads trimmed + merged	No. unique reads* mapped to ref.	% Ref. covered > 1x	Average coverage of ref.
**HBV** **X65257**						
LM1	Distal femur	1041774	543601	4338	95.5	76.4
LM2	Rib with skin	2312150	1189513	5	5.3	0
LM3	Fronto-parietal bone with skin	1489660	762137	757	87.3	12.6
LM11	Thigh muscle	1453500	758346	59	37.3	0.8
LM13	Temporo-maxillary skin	1551240	811581	77	52.2	1.2
LM14	Leg skin	1393556	733735	14	16.6	0.2
LMBLK	Blank control	72	45	0	0	0
LM14a-3	Leg skin	4545730	2329320	33	0.3	0.5
LMBb-3	Extraction blank	3870968	2442592	0	0	0
LMLBb-3	Library blank	107554	67319	0	0	0
HLM1a (non-UDG)	Distal femur	3869248	1974742	4360	96.6	72.6
HLMBa (non-UDG)	Blank control	1202	661	0	0	0
Pooled for consensus		13111128	6773655	9610	96.6	163.8
**mtDNA** **rCRS**						
LM14a-2	Leg skin	5943268	2993715	320289	100	1014.8
LMBb-2	Extraction blank	518416	313909	181	30	1
LMLBb-2	Library blank	20704	11082	5	2	0
**VARV** **NC_001511**						
LM1b-1	Distal femur	112632	59112	0	0	0
LM14a-1	Leg skin	1476	864	0	0	0
LMLBb-1	Library blank	10	6	0	0	0

*‘Unique reads’ refer to those merged or properly paired with a minimum length of 30bp.

**Fig 2:**
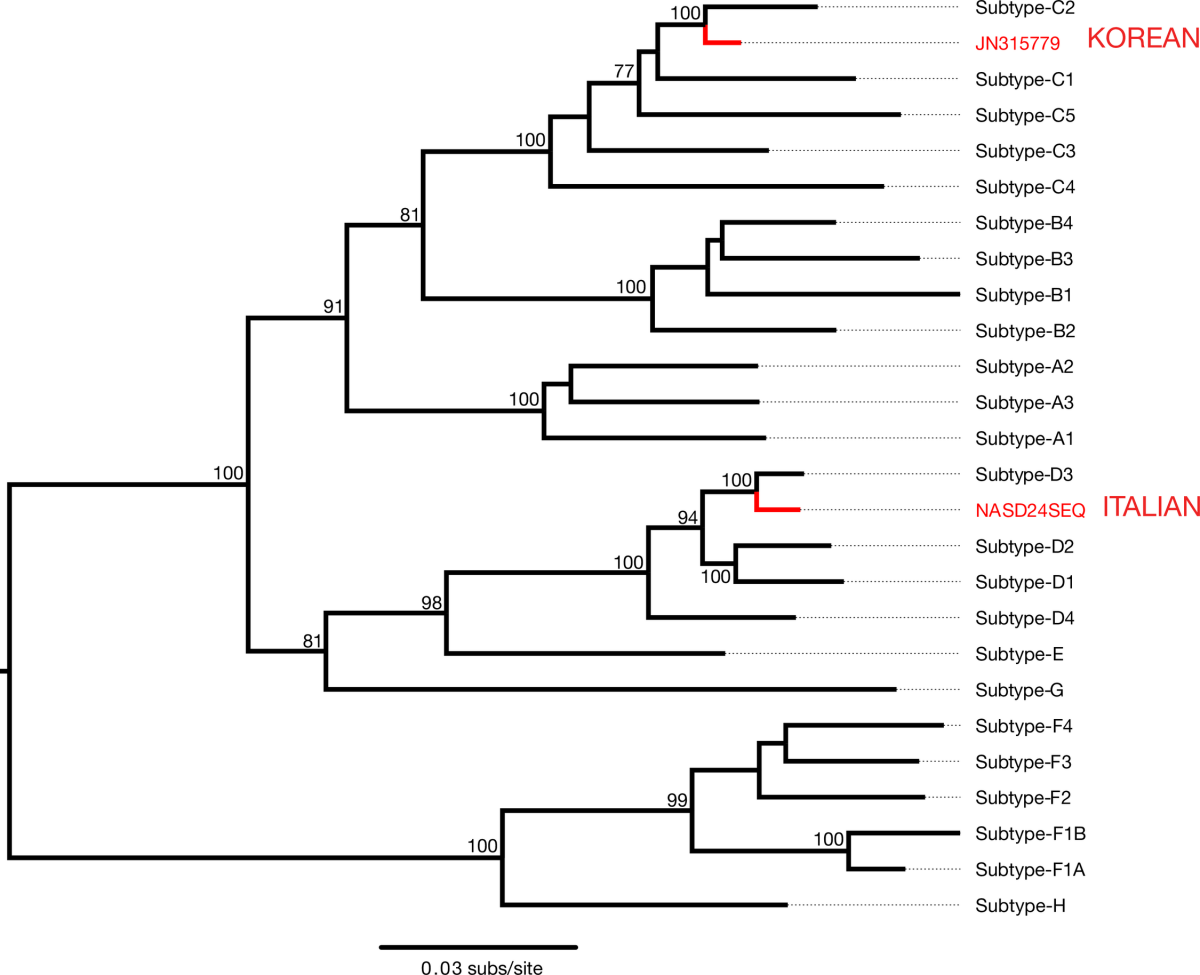
Phylogenetic analysis reveals that the HBV sequence reads from NASD24 are from a subgenotype D3 virus. Maximum likelihood phylogeny of HBV sequences representative of the full genotypic diversity. Tip labels indicate viral genotype. The draft genome from HBV reads from NASD24 (Italian Mummy) and the JN315779 sequence (Korean Mummy) are colored in red. Nodes with bootstrap support above 70% are displayed. All horizontal branch lengths are scaled according to the number of nucleotide substitutions per site and the tree is mid-point rooted for clarity only.

We first mapped all next-generation sequencing reads to the HBV reference genome (GenBank accession number NC_003977) using a dedicated aDNA pipeline [8] (Table 2, S1 Table). Using BLAST, as well as an initial phylogenetic analysis of a sequence data set representing the genotypic diversity of HBV (Fig 2, S2 Table), the consensus of the draft HBV genome was identified as a subgenotype D3 virus. This subgenotype has a broad global distribution and is common in the Mediterranean region, including Italy [30]. To ensure a proper consensus we remapped all reads to a subgenotype D3 HBV (GenBank accession number X65257). In addition we mapped all reads to the revised Cambridge reference sequence (rCRS) for human mtDNA (GenBank accession number NC_012920 [45]) and identified the consensus as haplogroup U5a1b (S3 Fig). Haplogroup U5 is common in European populations and U5a1b is most commonly found in Eastern European populations but is also frequently seen in the Mediterranean region, including in Greek, Italian, Portuguese and Spanish populations [46].

The HBV genome used in all evolutionary analyses was assembled after pooling all the reads from the rib with skin, fronto-parietal bone with skin, thigh muscle, temporo-maxillary skin, leg skin, and the distal femur both with and without UDG treatment. This consensus genome is 3,182 nt in length and with a gap in the genome of 177 nt (positions 1427–1603) near the 5’ end of the X ORF and in the region of overlap with the polymerase ORF (S2 Fig). This gap is likely due to low bait coverage because of locally high G/C strand imbalance affecting oligo production, rather than the existence of a true biological gap due to a deletion. The first 5 nucleotides of the sequence mapped to X65257 were marked as ambiguous, as were the last 36.

**Fig 3:**
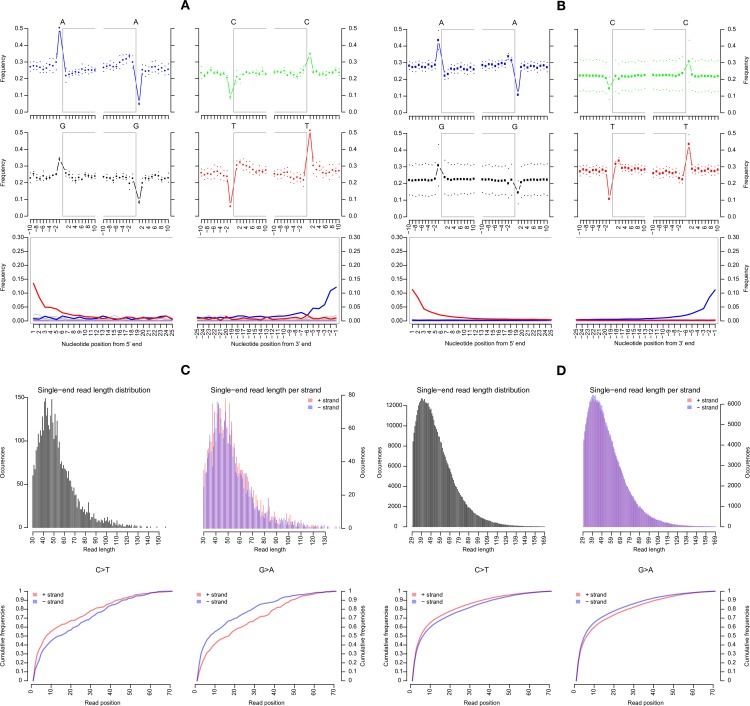
Damage patterns of NASD24 HBV and mtDNA sequenced reads. Next-generation sequencing reads from non-UDG treated distal femur extraction (LM1) were analyzed for nucleotide deamination patterns following removal of reads less than 30 nt in length and after mapping of remaining reads. The upper plots display the nucleotide base frequency with the grey box indicating frequency within the read. The bottom plots show the position’s specific substitutions from the 5’ (left) and the 3’ end (right) of each fragment. (A) Deamination plots for processed reads from NASD24 LM1 enriched for HBV and as mapped to HBV reference sequence X65257. (B) Deamination plots for processed reads from NASD24 LM1 enriched for human mtDNA and as mapped to mtDNA reference sequence NC_012920. (C) Fragment length distribution plot for HBV reads. (D) Fragment length distribution plot for mtDNA reads.

A damage analysis of the mapped reads (using the mapDamage 2.0 program [47]) from the subsamples with and without UDG treatment revealed an authentic post-mortem damage pattern, as expected for an ancient sample (Fig 3, S4 Fig). If deamination had occurred within the lifespan of an infected host (for instance, as a result of deamination induced by human enzyme APOBEC–known to induce deamination in certain viruses), then we would expect to have seen deamination spread more evenly throughout the viral reads. Instead, deamination has preferably occurred at the termini of DNA fragments as seen in the non-UDG treated subsamples (Fig 3A). As nearly identical patterns were observed with the mitochondrial reads, this suggests to us that the HBV DNA is more likely to be of the same age as the mtDNA than it is to be derived from a recent contaminant (Fig 3B). As expected, following treatment with UDG, viral reads do not display the deamination pattern, suggesting that the UDG did indeed remove uracils from the DNA fragments (S4 Fig) [48].

### Attempts to enrich VARV DNA

As it has been previously been suggested that the rash observed in this mummy is the direct result of a smallpox infection [43], we enriched for VARV using an in-solution bait set previously published [8]. Importantly, we were unable to find a single significant read mapping to the VARV genome (S1 Table). Although we cannot conclusively exclude the presence of VARV in this sample, this result does not lend credence to the presence of any VARV DNA when compared to the successful enrichment of HBV DNA, unless VARV is far more sensitive to degradation post mortem than HBV is, which does not agree with our recent success at enrichment of VARV from a Lithuanian child mummy [8].

### Evolutionary analysis of Italian HBV aDNA

We compiled two primary data sets representing (a) the full genotypic diversity of HBV and (b) that of D genotype alone (Table 3). These consisted of publicly available HBV sequence data from GenBank with the additions of the HBV genome newly sequenced here (NASD24SEQ) and that previously obtained from a Korean mummy (JN315779) (S2 Table). No evidence of recombination was found within either ancient sequence. While there was evidence for recombination in some modern sequences, equivalent results were found in evolutionary analyses conducted with and without these sequences, indicating that recombination has not had a major impact on the phylogenetic results presented here.

**Table 3:** HBV data sets. All individual data sets were compiled from a larger overall data set of whole-genome HBV sequences that included a collection date (1963–2014). See Methods for a more comprehensive description of individual data sets.

Name	Sampling range (year)	Size (no. sequences)	Description
a	1963–2013	135	Random subsample of available HBV genomes
a-i	1568–2013	136	Subset a with the addition of NASD24SEQ
a-ii	1568–2013	137	Subset a-i with the addition of JN315779
b	1975–2013	62	Random subsample of all available D genotype sequences
b-i	1568–2013	55	Subset b with the addition of NASD24SEQ

**Fig 4:**
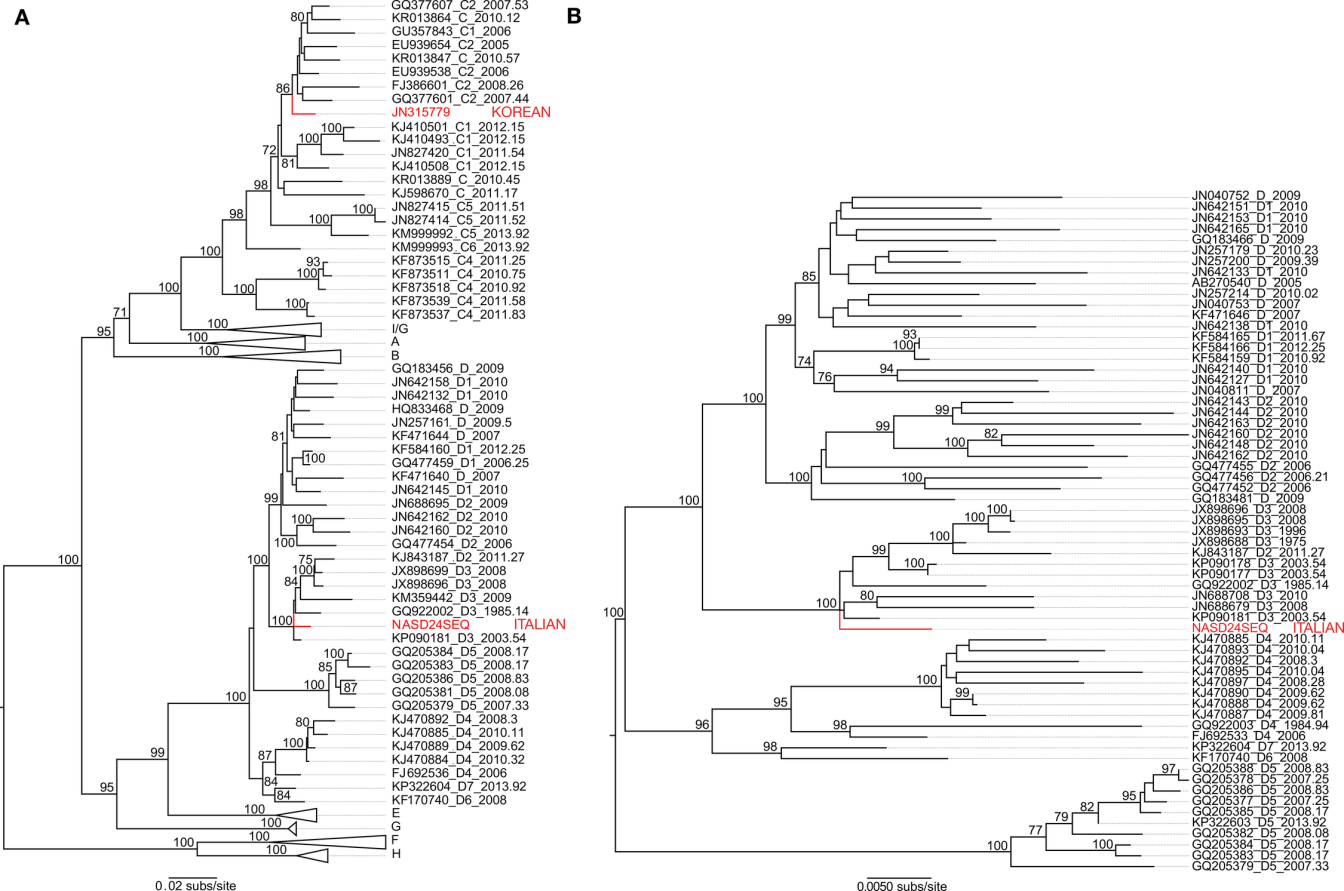
Phylogenetic analysis of ancient and modern HBV sequences. Tip labels on the phylogenies include the GenBank accession number followed by genotype and subgenotype information and year of sampling (day and month information is encoded into decimal form). NASD24SEQ and JN315779 are colored in red. Nodes receiving bootstrap support above 70% are displayed. All horizontal branch lengths are scaled according to the number of nucleotide substitutions per site and the tree is mid-point rooted for clarity only. (A) Shows the analysis of subset a-ii, including the modern HBV subset with both ancient sequences. (B) Shows the analysis of subset b-i, including only the modern D genotype subset with NASD24SEQ.

Maximum likelihood phylogenetic analysis revealed that the Italian sequence (NASD24SEQ) and the previously published Korean sequence (JN315779) fall within the genetic diversity of modern HBV [12]. Specifically, in phylogenetic analysis of HBV sequences representative of all genotypes (data set a-ii), NASD24SEQ grouped with modern HBV sequences of the D3 subgenotype collected between 1985 and 2008 with 100% bootstrap support, falling on the branch separating D3 from the D1 and D2 subtypes (Fig 4). Similarly, JN315779 fell within the genetic diversity of HBV genotype C, on the branch separating subgenotype C2 (Fig 4).

Importantly, NASD24SEQ occupied similar phylogenetic positions within subgenotype D3 when phylogenies were inferred separately for the overlapping and non-overlapping regions of the HBV genome, as well as for the polymerase ORF alone (S5 Fig). Hence, there is no evidence that the grouping of NASD24SEQ with modern subgenotype D3 sequences is a function of genome overlap.

The sequence recovered from this 16^th^ century Italian mummy therefore occupies a paradoxical phylogenetic position: although it exhibits legitimate signs of DNA damage, consistent with both the pattern seen in the mitochondrial reads and an ancient origin, it clusters closely with modern HBV sequences, as might be expected if it were a recent contaminant. If the NASD24SEQ sequence is *bona fide*, then the only reasonable explanation is that our data set representing the last 450 years of HBV evolution is of insufficient duration to exhibit temporal structure, in turn implying that HBV has a long evolutionary history in humans with ancient diversification times of the different viral subtypes. To test this hypothesis, we performed a detailed analysis of HBV evolutionary dynamics, focused on addressing whether the evolution of this virus presents sufficient temporal structure for molecular clock dating.

To help determine the veracity of our ancient HBV sequence, we performed a series of analyses using both root-to-tip regression [49] as well as those within a Bayesian framework [50]. These analyses employed various calibrations, including collection dates for modern samples, radiocarbon-dating estimates of ancient samples, and viral co-divergence with human population migration.

**Fig 5:**
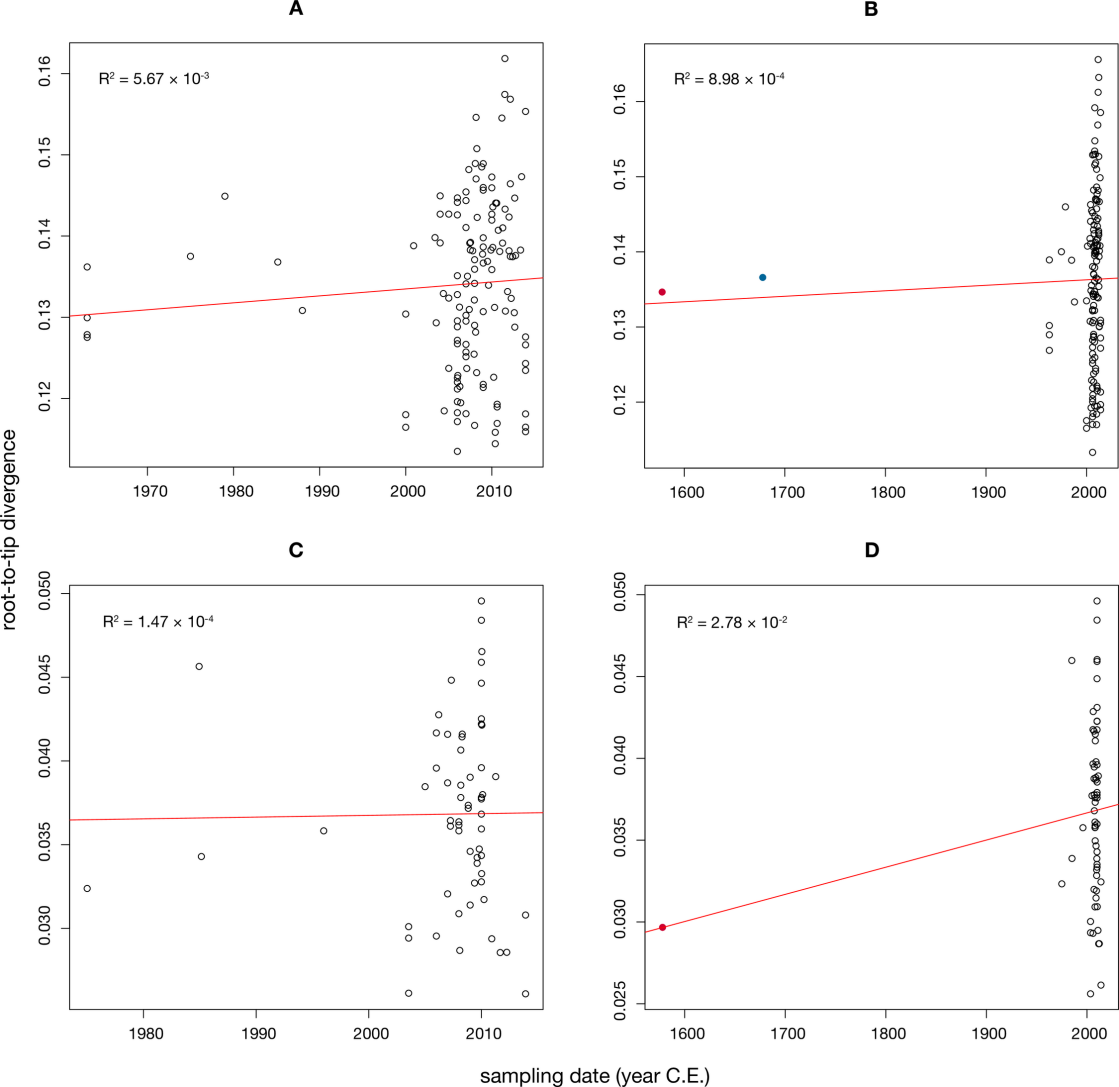
Root-to-tip regression analyses of temporal structure. Plots of the root-to-tip genetic distance against sampling time (year) are shown for phylogenies estimated from alignments of random subsamples of whole HBV diversity (subset a) or only D genotype diversity (subset b). Each dot represents a sequence, with black dots representing modern samples while that for NASD24SEQ is colored in red and that for JN315779 is colored in blue. R^2^ values are given as an indicator of the degree to which evolution has been clock-like. The central line corresponds to the regression line and the outer lines to the 95% confidence intervals. Regression analysis for other data sets are shown in S6 Fig. (A) Modern sequences only (subset a). (B) All modern and ancient HBV sequences (subset a-ii). (C) Modern genotype D sequences (subset b). (D) Modern genotype D sequences with NASD24SEQ (subset b-i).

**Fig 6:**
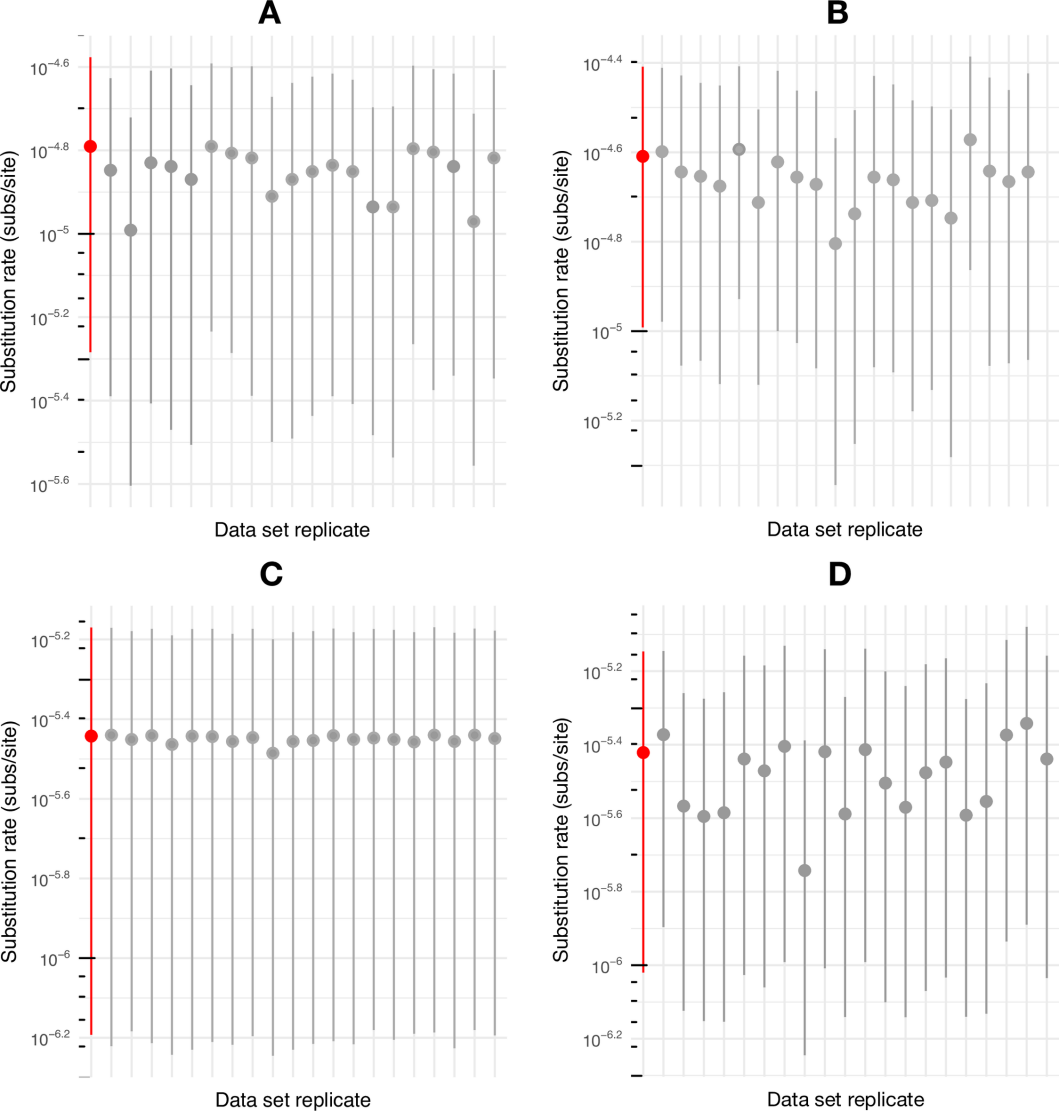
Date randomization test. Estimates of the nucleotide substitution rate (subs/site/year) for the date-randomized subsets of HBV sequences reflecting either all genotypes (a) or only D genotype (b). The y-axis indicates the substitution rate (log_10_ scale) and the x-axis shows different randomizations of each data set, with the non-randomized data set (i.e. with dates correctly assigned to sequences) colored in red. The circles represent the mean rate estimates and the error bars show the 95% credible intervals. (A) Modern HBV samples only (subset a). (B) All modern and the ancient HBV samples (subset a-ii). (C) All modern genotype D samples (subset b). (D) All modern genotype D samples with NASD24SEQ included (subset b-i).

Data sets must possess temporal structure for tip-dated analyses to be informative [51]. We first assessed for temporal structure using a regression of root-to-tip genetic distances against year of sampling [49]. Strikingly, neither of the primary data sets (subsets a and b), with or without inclusion of ancient sequences, showed evidence of any temporal structure, with R^2^ values of 8.98 × 10^−4^ (a-ii) and 2.78 × 10^−2^ (b-i), respectively (Fig 5), as was true of the other genomic data sets (S6 Fig). Similarly, no temporal structure was observed in the D3 subgenotype, both without (R^2^ = 2.85 × 10^−2^) and with NASD24SEQ (R^2^ = 2.90 × 10^−2^) (S7 Fig). To further assess the extent of temporal structure, we employed a Bayesian date-randomization test in which the nucleotide substitution rate is estimated using the correct sampling dates (see below), and the analysis then repeated 20 times on data sets in which the sampling dates have been randomized among the sequences [52]. Notably, for both ancient sequences (NASD24SEQ and JN315779), the 95% higher posterior density interval (HPD) of the rate overlapped between the true and randomized data for both the modern and complete (including ancient) data sets (Fig 6 and S8 Fig). This indicates that there is insufficient temporal structure in HBV to performed tip-date-based analyses of evolutionary dynamics, even when including sequences that date to the 16^th^ century.

Because of the lack of temporal structure from tip-dated calibrations, we next specified an informative prior distribution on the clock rate using a previous estimate of the long-term substitution rate of HBV at 2.2 × 10^−6^ subs/site/year [24]. The aim here was to estimate the ages of NASD24SEQ and JN315779 and determine whether these estimates matched the dates inferred from radiocarbon dating of the mummies and tomb materials. To this end, we tested (i) a prior of 2010 on the ages of both modern and ancient samples, such that no true sampling dates were considered, (ii) a uniform prior on the age of the ancient samples with a lower bound of 0.0 and an upper bound of 10,000 years before present, and (iii) a normal prior matching the radiocarbon dates. For both the ‘modern’ prior of 2010 and the uniform prior calibrations we expect that the posterior will differ from the prior if the rate calibration and the molecular sequence data are informative about the ages of the samples. Importantly, the inclusion of this long-term substitution rate as a clock calibration resulted in estimated ages for the ancient samples that were very similar to each of the priors tested in each case (S9A Fig). Hence, the molecular sequence data and rate calibration do not have sufficient information to estimate the age of these sequences.

Similarly, we attempted to estimate the sampling times of NASD24SEQ and JN315779 under the assumption that HBV has co-diverged with human populations. Internal node calibrations can be more informative than tip calibrations when no temporal structure can be ascertained using sampling dates [51, 53]. Accordingly, we used the same calibration scheme as Paraskevis *et al*. 2015, in which human migration dates were used to specify the prior distributions of ages on nodes from HBV subgenotypes found in endemic populations [24]. Our analysis using subset a-ii yielded a mean rate estimate of 6.84 × 10^−6^ subs/site/year (95% HPD: 4.46 × 10^−6^ to 9.26 ×10^−6^ subs/site/year), which is considerably lower than some previous estimates [25]. Critically, however, the age estimates for the ancient samples again matched the uniform prior distributions (S9B Fig). Therefore, even with internal node calibrations, the sequence data and calibrations were not sufficiently informative to estimate the age of these viral sequences.

**Fig 7:**
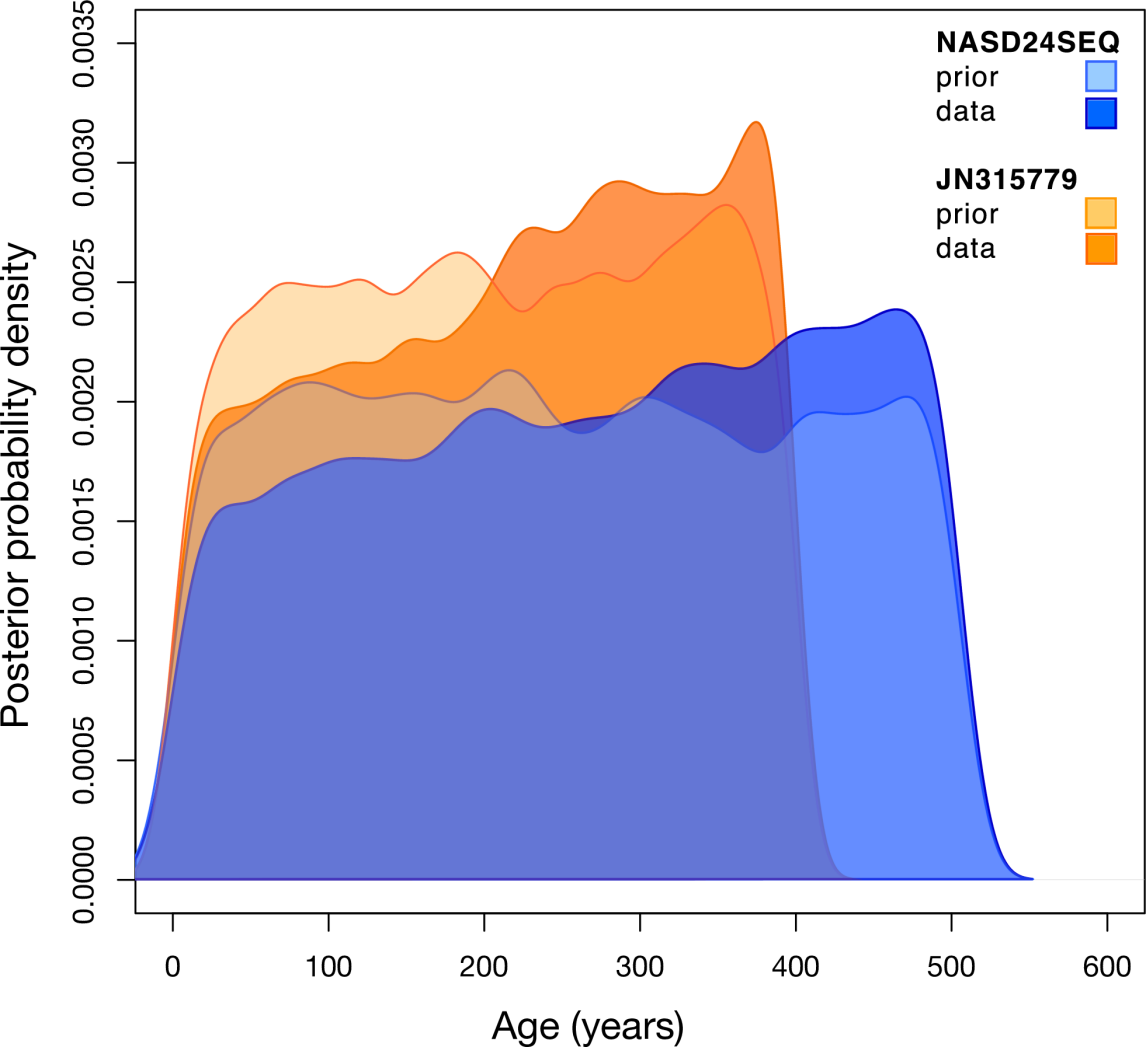
Comparison of prior and posterior probability distribution estimates for the age of ancient HBV samples NASD24SEQ and JN315779. Histogram showing the probability density estimation distributions for the Bayesian analysis of NASD24SEQ and JN315779 both with and without sequence data from subset a-ii, using a normal prior approximating the radiocarbon dates for the samples and with the internal calibration scheme using the estimation of entry into the Americas from Llamas et al. 2016 [55] to calibrate the node separating genotypes F and H.

Finally, we employed a second set of internal node calibrations in which the node separating the F and H genotypes from the rest of the HBV tree had a normal prior with a mean of 16,000 years and a standard deviation of 1000 years. This follows a study that estimated that humans may have entered the Americas about 16,000 years ago, a more specific date than that estimated in earlier papers [54]. Using this more precise calibration, our analysis resulted in a mean rate estimate of 4.57 ×10^−6^ subs/site/year (95% HPD: 2.62 × 10^−6^ to 6.96 ×10^−6^ subs/site/year) similar to that obtained from the node calibrations employed above. The mean ages of the ancient samples were accordingly estimated at 214 years for JN315779 (95% HPD: 23 to 398) and 276 years (95% HPD: 29, 505) for NASD24SEQ (Fig 7). However, due to the very wide uncertainty in these estimates, which again closely resemble the prior distributions, these results do not provide posterior estimates that are conclusive on the age of the HBV samples.

## Discussion

We have enriched and sequenced a complete HBV genome from the remains of a mummified child estimated to have died in 1569 CE ± 60 years [39]. The cytosine deamination patterns occurring preferentially at termini in both the viral and mitochondrial DNA fragments support the ancient authenticity of these sequences. We have also subtyped the mitochondrial DNA from the mummy to haplogroup U5a1b, a common European haplogroup [46], and the HBV to genotype D, a genotype predominant in the Mediterranean region today [30]. The nearly identical fragment length distributions, deamination patterns and same geographically recovered haplotypes (mitochondrial and HBV) argue for the authenticity of the sequences. The identification of consistent HBV reads in multiple (5) tissue samples (distal femur, fronto-parietal bone with skin, thigh muscle, temporo-maxillary skin and leg skin; Table 2), suggests that the virus is distributed throughout the mummy and not in one location, as might be expected with contamination. Further, other mummies from the same site, excavated at the same time and processed in the same facilities, did not show any HBV reads in shotgun sequencing data. Thus, if the mummy was contaminated, it was specific to this one sample alone. Although hepadnaviruses like HBV exhibit strong tropism for liver cells (hepatocytes), hepadnaviral DNA has been shown to exist in other somatic cells, including mononuclear cells, which are protected by the hydroxalite matrix of the bone [55–57]. HBV particles produced in the bone marrow and protected by the matrix may explain why we recovered the majority of our viral sequences from a femur sample. DNA isolated from ancient bone matrix has also been shown to be better preserved and less damaged than that recovered from corresponding soft tissue from the same remains [58].

Many reports of ancient epidemics and other disease outbreaks have relied upon historical reporting and paleopathological studies of human remains. Recent advances, including next generation sequencing technology [5] and DNA enrichment methods [6], now allow recovery of ancient nucleotide sequences from these remains and the genetic verification of the pathogens responsible for disease, as well as the identification of pathogens undetectable by other means. Our study provides a strong argument for this latter approach, as mummy NASD24 was originally reported to have been infected with smallpox [40, 43]; crucially, however, shotgun sequencing following enrichment for VARV (S1 Table) and SEM analysis (S1 Fig) revealed no evidence of VARV in this mummy. This is particularly surprising given previous results in which electron microscopy studies and immunostaining indicated the presence of VARV particles in these samples [43]. Given our results, a new interpretation is that the child was not suffering from smallpox at the time of death, but rather Gianotti-Crosti syndrome caused by HBV infection [59]. Gianotti-Crosti syndrome is a rare clinical outcome of HBV that presents as a papular acrodermatitis in children between 2 and 6 years old [59]. This, in turn, illuminates the power of aDNA in providing definitive evidence or clarifying retrospective diagnoses, where etiology may be uncertain and morphology complicated for key type specimens that provide critical time points for the origins or presence of specific pathogens (e.g. smallpox).

Despite the multiple streams of evidence supporting an ancient origin of NASD24SEQ, the results of the evolutionary analysis are less straightforward. In particular, our phylogenetic analysis reveals a close relationship between NASD24SEQ and modern D genotype sequences, as would be expected if the sequence were a modern (1980s) contaminant, and we note the same phenomenon with the Korean mummy sequence thought to date from the 17^th^ C [12]. Importantly, however, data sets representing only modern HBV sequences, sampled over 50 years to the present, did not display discernible temporal structure. Clearly, without temporal structure we cannot accurately estimate the age of the ancient sequences using phylogenetic methods. Hence, the apparently paradoxical phylogenetic position of NASD24SEQ cannot automatically be taken to mean that this genome is a modern contaminant. In turn, if NASD24SEQ is indeed from the 16^th^ century, then this phylogenetic pattern indicates that the diversification of the HBV genotypes occurred prior to 1500 and that any subsequent accumulation of diversity was either lost through strong purifying selection or masked by multiple substitutions.

Our analyses of both modern and ancient HBV samples returned results consistent with the absence of temporal structure, not only within the full diversity of HBV but also within the D genotype and D3 subgenotype. Given that the genomic structure of HBV is likely to result in strong selective constraints, a likely explanation for our results is that many of the mutations that arise in the short-term, such as within chronically infected hosts or along single chains of transmission, are non-synonymous and eventually removed from the HBV population by purifying selection, yet artificially inflating evolutionary rates over this sampling period [60]. Support for this hypothesis comes from short-term studies of HBV evolution in which rates of evolutionary change are greater than those estimated from longer-term studies [24, 25].

On balance, our analysis suggests that our HBV sequence is authentically 16^th^ century and that no temporal structure is observable in over 450 years of HBV evolution. As such, these results have a number of important implications for the study of HBV evolution. In particular, such a phylogenetic pattern implies that the currently circulating viral genotypes must have been associated with their specific host populations long before the 16^th^ century, and hence supports a long association of HBV with human populations. In addition, the lack of temporal structure means that it is not possible to use molecular clock methods to reliably date HBV evolution over the time span of genome sequences currently available.

## Materials and methods

### Sample history

Exploration of the coffin and the autopsy of the unidentified two-year old mummy (NASD24) was completed as part of a larger study, conducted between 1984 and 1987, of mummies from the sacristy of the Basilica of Saint Domenico Maggiore in Naples, Italy. Autopsies were performed for all mummies by paleopathologists wearing sterile surgical coats, sterile latex gloves, sterile masks, headdresses and overshoes. Details of this initial investigation have been reported previously [40].

The samples used for aDNA sequencing were collected during the initial autopsies, and these samples were stored in sealed, sterile plastic bags. The sample bags were first opened in 1985 for a preliminary paleopathological examination that suggested a viral agent, thought to be smallpox, as the likely cause of an apparent skin rash [40]. The samples were handled again in 1986 for examination by immune-electron microscopy [43]. After this, the samples remained in sterile storage before subsamples were removed and sent to the McMaster Ancient DNA Centre at McMaster University (Hamilton, Ontario, Canada) in 2013.

### Ethics approval

Ethical approval to work on this mummy sample was granted to Dr. Fornaciari by the Supervisor for the Artists and Historians of Campania in 1984.

### aDNA library preparation, enrichment and sequencing

Eight subsamples of 75–125 mg of organic matter were excised from samples of various body parts of NASD24 in a dedicated cleanroom facility at the McMaster Ancient DNA Centre (Table 1). Tissue samples were cut into small pieces using a scalpel and bone material crushed into powder. These samples were then demineralized, digested, and extracted according to previously published protocols [44]. In brief, samples were demineralised using 1.5 mL of EDTA (0.5 M, pH 8.0) before being incubated at room temperature for 24 hours with rotation at 1000 rpm. Samples were then digested using a 1.5 mL proteinase K solution and incubated at 55°C for 6 hours with rotation at 1000 rpm. The supernatant from the demineralization and digestion steps was subjected to organic extraction using a modified phenol-chloroform-isoamyl (PCl) alcohol protocol. This means that 0.75 mL of PCl (25:24:1) was added to the demineralization/digestion supernatant, which was then vortexed, and spun via centrifuge (4000×g) for 20 minutes. The aqueous phase was transferred to a fresh tube and a further 0.75 mL of chloroform added. The solution was mixed and spun via centrifuge (4000×g) for 10 minutes. The aqueous phase was collected and concentrated using an Amicon Ultra 0.5 mL 30 kDa filter. This concentrated solution was purified over a MinElute column (Qiagen, Hilden, Germany) according to the manufacturer’s instructions, and eluted in 10 μL of 0.1 TE with 0.05% Tween-20. Reagent blanks were introduced at each step and processed alongside the samples.

A library from the distal femoral sample of NASD24 was prepared according to a previously published protocol [61] that was modified to include an overnight ligation and with an input volume of 5 μL. Double indexing was performed using KAPA SYBR FAST (Kapa Biosystems) for 8 cycles of indexing amplification [62]. The library and blanks were enriched using two rounds of in-solution capture baits targeting HBV and the human mitochondrial genome (in separate reactions) according to the manufacturer’s instructions (Mycrorarray, MyBaits) with recommended aDNA modifications. Baits, of 80nt in length with 4x tiling density (10nt flexible spacing), were designed based on the sequences of 5,230 HBV sequences, representing all major viral subtyptes. Template input was 5 μL for each reaction and bait concentrations were 100 ng per reaction using the in-solution bait mix targeting HBV and 50 ng per reaction for that targeting human mtDNA. Target genetic material was reamplified for 12 cycles both between and after rounds of enrichment. The HBV-enriched library generated 1,934,624 clusters (3,869,248 raw reads) and the human mtDNA-enriched library generated 2,844,500 clusters (5,689,000 raw reads) on an Illumina HiSeq 1500 at the Farncombe Metagenomics Facility (McMaster University, Hamilton Ontario, Canada).

### Sequence data trimming, analysis and assemblies

Reads were demultiplexed using CASAVA-1.8.2 (Illumina, San Diego, California), then adapters were trimmed and reads merged using leeHom [63] with aDNA specific settings (—ancientdna). These processed reads were mapped to an appropriate reference genome (HBV genotype D3, GenBank accession X65257; revised Cambridge Reference Sequence for human mtDNA, GenBank accession number, NC_012920) using a network-aware version of the Burrows-Wheeler Aligner [64] (https://bitbucket.org/ustenzel/network-aware-bwa) with distance, gap and seed parameters as previously described [8]. Duplicates were removed based on 5’ and 3’ positions (https://bitbucket.org/ustenzel/biohazard). Reads shorter than 30 base pairs and with mapping quality less than 30 were removed using Samtools [65]. The resulting BAM files were processed using mapDamage 2.0 on default settings with plotting and statistical estimation [47]. Haplogrep v2.1.0 [66] using PhyloTree Build 17 [67] was used to identify the haplogroup of the mtDNA as U5a1b. The complete genome sequence of NASD24 has been submitted to GenBank and assigned accession number MG585269.

### Data set assembly

We analyzed the ancient HBV sequenced in this study in the context of modern whole-genomes of HBV. To this end we downloaded all human HBV genomes from GenBank that were over 3,000 nt in length and for which the year of sampling was available (all date information, including month and day, if available, was converted into decimal format). This initial GenBank data set comprised 3,696 sequences sampled between 1963 and 2015 (S2 Table). Sequences were aligned with the MAFFT v7 program using the FFT-NS-1 routine [68] to visually check for obvious errors in database labeling.

For initial genotypic subtyping, one representative of each HBV subtype was selected (S3 Table). This data set included the purportedly ancient HBV sequence previously obtained from a 17^th^ century Korean mummy (GenBank accession number JN315779; radiocarbon-dated to 1682 with an error range of 1612–1752 [12]). The subtype of NASD24SEQ was inferred from maximum likelihood (ML) phylogenetic trees estimated using PhyML v3.0 [69] with the GTR+Г_4_ model of nucleotide substitution and employing SPR branch-swapping, with nodal support assessed by conducting 1000 non-parametric bootstrap replicates.

Following this, a random subsample of HBV sequences was taken using the *Ape* package in R [70]. Specifically, we sampled five representatives of each genotype and subtype, or the maximum number available if this was not five. This produced a data set of 135 sequences sampled between 1963–2013 which we refer to as subset a (Table 3, S4 Table). We then added the ancient Italian HBV sequence NASD24SEQ to subset a to make data set a-i with n = 136. A third subset was built by adding the ancient Korean HBV sequence JN315779 to a-i to make subset a-ii with n = 137. We next randomly sampled only D genotype sequences from the initial GenBank data set to form subset b with n = 64 (S5 Table). To this we added NASD24SEQ, generating subset b-i with n = 65. We aligned the nucleotide sequences in each subset using the L-INS-i routine in MAFFT v7.

### Recombination analysis

The RDP, GENECOV, and MAXCHI methods available within the RDP v4 package [71], with a window size of 100 nt (and default parameters), were used to analyze each subset for recombination. If at least two methods detected recombinant regions in a sequence, then we removed the region of recombination from the alignment.

### Phylogenetic analysis

Following removal of recombinant regions, we inferred phylogenetic trees on each subset (of a and b) again using the ML method in PhyML v3.0 [69] with the GTR+Γ_4_ model of nucleotide substitution and employing SPR branch-swapping, and with nodal support assessed by conducting 1000 non-parametric bootstrap replicates.

### Assessing the temporal structure of HBV

We conducted a range of analyses to assess the extent of temporal structure in the data sets and to estimate the rate and time-scale of HBV evolution. To initially verify the temporal structure in the data we conducted regressions of root-to-tip genetic distance as a function of the sampling time (year) using TempEst v0.1 [49]. We then conducted a date-randomization test [52]. This involved analyzing the data using the Bayesian method implemented in BEAST v1.8.3 [50] under a lognormal relaxed clock model [72] and assuming a constant population size, with 20 replicates in which the sampling dates were randomized among the sequences. The HBV data were considered to have temporal structure if the mean rate estimate and 95% HPD intervals were not contained within the 95% HPD of any of estimates resulting from the randomized data sets [52]. All analyses were run with an Markov chain Monte Carlo chain length of 10^7^ steps with samples from the posterior distribution drawn every 2 × 10^3^ steps. After discarding the first 10% of steps as burn-in, we assessed sufficient sampling from the posterior by visually inspecting the trace file and ensuring that the effective sample sizes for all parameters were at least 200.

We also performed more detailed Bayesian estimates of evolutionary dynamics. One method of validating the ages of ancient samples is to specify uninformative prior distributions for these and test if the tip dates or an informative rate give the postulated ages. Specifically, if the sequence data and calibrations are informative, the posterior should consist of a narrow distribution that includes the true sampling time of the ancient samples [73]. However, if the prior and posterior distributions for the ages of the ancient samples are the same, then the data are considered to have insufficient information to estimate the ages of these samples. We conducted these analyses by setting uniform distributions for the two ancient samples between 0 and 10^7^ with a mean of 10^5^. For the Korean sample we set a uniform prior with upper and lower bounds of 400 and 0, whereas for the Italian sample we used 507 and 0, with the maximum values in both cases reflecting their presumed sampling date. We also conducted analyses in which, for the Italian sample, we set a normal truncated prior distribution with the upper and lower values at 507 and 387, and mean of 447 and standard deviation of 10, whereas for the Korean sample we used 400 and 260 for the bounds, and 330 and 35 for the mean and standard deviation, respectively. These numbers are based on the radiocarbon dating analysis, with the upper and lower values reflecting the error margins.

We employed three calibration strategies for these analyses. (i) First, we used the sampling times of the modern samples, but with a uniform prior for the rate bounded between 0 and 1. (ii) Second, we assumed that all the modern samples were contemporaneous, and specified internal node calibrations, corresponding to those used by Paraskevis *et al*. (2015) and Llamas *et al*. (2016) [36, 54] and assuming that the spread of HBV corresponds to that of early human populations [24, 36]. These consisted of setting normal priors for the time of the most recent common ancestor (tMRCA) of F and H genotypes at 16,000 years, with a standard deviation of 1000, setting the tMRCA for subgenotype B6 at 3500 years with a standard deviation of 3000, and setting the tMRCA for subgenotype D4 at 8500 years with a standard deviation of 3500. (iii) Finally, we calibrated the molecular clock by specifying an informative prior for the mean (long-term) substitution rate of HBV based on the estimate by Paraskevis *et al*. (2013) [24]. Accordingly, the prior was in the form of a normal truncated prior with mean of 2.2 × 10^−6^ sub/site/year, standard deviation of 5 × 10^−7^, and lower and upper bound of 1.5 × 10^−6^ and 3 × 10^−6^, respectively. We again used the GTR+Γ_4_ nucleotide substitution model for these analyses, although a codon-partitioned HKY model was also considered within the internally calibrated analysis.

## Supporting information

S1 Fig SEM images displaying morphology and size of putative viral particles in the thigh muscle of NASD24. (PDF)

S2 Fig NASD24SEQ consensus sequence as constructed from mapping next-generation sequencing reads from NASD24 LM1 to HBV subgenotype D3 sequence X65257. Genomic organization of overlapping open reading frames and approximate location of single-stranded portion of plus strand are indicated, as well as the relative GC (blue) to AT (green) content of the genome of X65257 and the likely location of CpG islands (light green).
(PDF)

S3 Fig Graphical phylogenetic tree representation of mtDNA classification results from Haplogrep run with Phylotree Build 17 for mtDNA reads from LM01 library of NASD-24. (PDF)

S4 Fig Analysis of fragmentation and cytosine deamination patterns of HBV reads from the UDG-treated LM01 library. (PDF)

S5 Fig Maximum likelihood phylogenetic trees for subset a-i, polymerase ORF, non-overlapping regions and only-overlapping regions of HBV genomes in subset a-ii. (PDF)

S6 Fig Linear regression analyses of HBV polymerase of subset a-i, as well as the polymerase, non-overlapping regions and only-overlapping regions of genomes in subset a-ii. (PDF)

S7 Fig Root-to-tip regression analyses of temporal structure in the D3 subgenotype. (A) Displays the D3 subgenotype. (B) Displays the D3 subgenotype with the addition of NASD24SEQ.
(PDF)

S8 Fig Date randomization tests of subset a-i, and the polymerase ORF, non-overlapping regions and only-overlapping regions of genomes in subset a-ii. (PDF)

S9 Fig Age estimate results for further calibration schemes and tests using BEAST v1.8.3. Histogram showing the probability density estimation distributions for the Bayesian analysis of NASD24SEQ and JN315779 both with and without sequence data from subset a-ii and with the internal calibration scheme using the estimation of entry into the Americas from Llamas et al. 2016 [55] to calibrate the node separating genotypes F and H. (A) With a uniform prior bounded by 0 and 1,000 years for the samples. (B) With a uniform prior bounded by 0 and 10,000 years.
(PDF)

S1 Table All tissue samples and shotgun and next-generation sequencing results, as well as results on mapping of reads to HBV subgenotype D3 reference X65257 and results of further analyses. (CSV)

S2 Table Accession numbers of all HBV sequences with collection data from Genbank and from which the data sets (a and b) used in this study were compiled. (TXT)

S3 Table Accession numbers of HBV sequences chosen to reflect each genotype and subgenotype and from relationships with which the subtype of NASD24SEQ was inferred following phylogenetic analysis. (CSV)

S4 Table Accession numbers of HBV sequences in data set a. Data set a comprises a random subsample of the publicly available HBV sequences on Genbank compiled such as to represent the full genotype diversity of HBV.
(CSV)

S5 Table Accession numbers of HBV sequences in data set b. Data set b comprises a random subsample of only D genotype HBV sequences compiled to represent the full subgenotypic diversity of D genotype.
(CSV)

S6 Table Accession numbers of D3 subgenotype sequences used for analysis, along with the accession number for the outgroup used to root this tree. (CSV)
