# Correction: The paradox of HBV evolution as revealed from a 16^th^ century mummy

**DOI:** 10.1371/journal.ppat.1006887

**Published:** 2018-02-09

**Authors:** Zoe Patterson Ross, Jennifer Klunk, Gino Fornaciari, Valentina Giuffra, Sebastian Duchêne, Ana T. Duggan, Debi Poinar, Mark W. Douglas, John-Sebastian Eden, Edward C. Holmes, Hendrik N. Poinar

There are multiple errors in the manuscript.

The authors inadvertently omit Dr. Caitlin Pepperell in their Acknowledgements section.

The correct Acknowledgements section should read: We thank Caitlin Pepperell and the McMaster aDNA Centre for comments on early versions of this work and for valuable insights into possible causes of the rash in the child mummy, Filip Braet for advice on interpretation of electron microscopy results, Simon Ho for advice on the molecular clock analysis, and Jemma Geoghegan for assistance with Fig 1. We also acknowledge the Sydney Informatics Hub and the University of Sydney HPC cluster Artemis for providing the HPC resources that contributed to the research results reported within this paper.

S1 Fig is omitted from the list of Supporting Information. As a result of this error, S2, S3, S4, S5, S6, S7, S8 and S9 Figs are incorrect. Please see the correct Supporting Figures here.

The Supporting information legends as they appear are correct:

## Supporting information

S1 Fig SEM images displaying morphology and size of putative viral particles in the thigh muscle of NASD24. (PDF)

S2 Fig NASD24SEQ consensus sequence as constructed from mapping next-generation sequencing reads from NASD24 LM1 to HBV subgenotype D3 sequence X65257. Genomic organization of overlapping open reading frames and approximate location of single-stranded portion of plus strand are indicated, as well as the relative GC (blue) to AT (green) content of the genome of X65257 and the likely location of CpG islands (light green).
(PDF)

S3 Fig Graphical phylogenetic tree representation of mtDNA classification results from Haplogrep run with Phylotree Build 17 for mtDNA reads from LM01 library of NASD-24. (PDF)

S4 Fig Analysis of fragmentation and cytosine deamination patterns of HBV reads from the UDG-treated LM01 library. (PDF)

S5 Fig Maximum likelihood phylogenetic trees for subset a-i, polymerase ORF, non-overlapping regions and only-overlapping regions of HBV genomes in subset a-ii. (PDF)

S6 Fig Linear regression analyses of HBV polymerase of subset a-i, as well as the polymerase, non-overlapping regions and only-overlapping regions of genomes in subset a-ii. (PDF)

S7 Fig Root-to-tip regression analyses of temporal structure in the D3 subgenotype. (A) Displays the D3 subgenotype. (B) Displays the D3 subgenotype with the addition of NASD24SEQ.
(PDF)

S8 Fig Date randomization tests of subset a-i, and the polymerase ORF, non-overlapping regions and only-overlapping regions of genomes in subset a-ii. (PDF)

S9 Fig Age estimate results for further calibration schemes and tests using BEAST v1.8.3. Histogram showing the probability density estimation distributions for the Bayesian analysis of NASD24SEQ and JN315779 both with and without sequence data from subset a-ii and with the internal calibration scheme using the estimation of entry into the Americas from Llamas et al. 2016 [55] to calibrate the node separating genotypes F and H. (A) With a uniform prior bounded by 0 and 1,000 years for the samples. (B) With a uniform prior bounded by 0 and 10,000 years.
(PDF)
