# Supplementary material for: The paradox of HBV evolution as revealed from a 16th century mummy
Source: PLoS Pathog. 2018 Jan 4;14(1):e1006750. doi: 10.1371/journal.ppat.1006750 (PMC5754119; doi:10.1371/journal.ppat.1006750)
Supplement: S1 Fig — (PDF) [file ppat.1006750.s001.pdf]

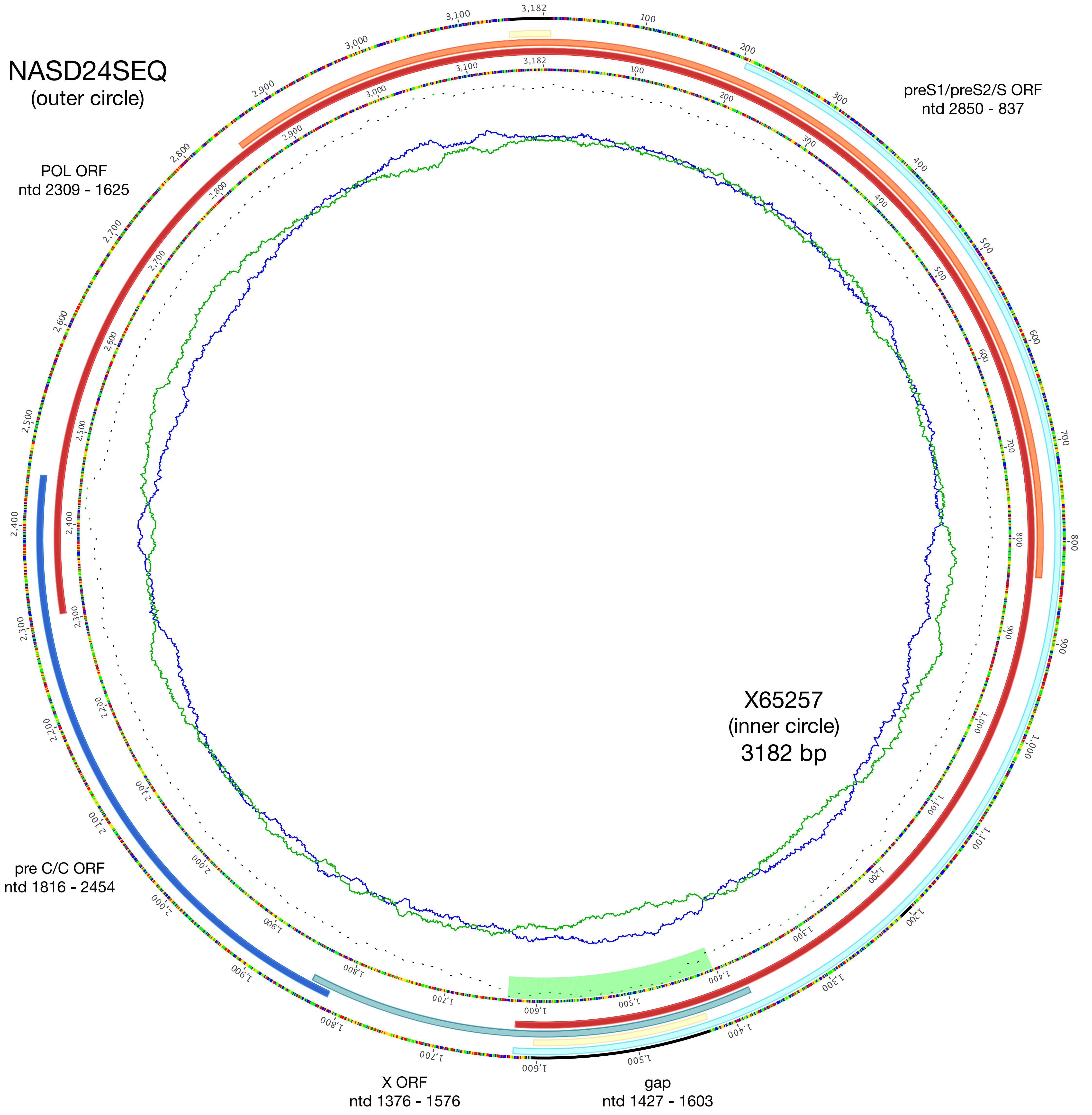
NASD24SEQ
(outer circle)
X65257
(inner circle)
3182 bp
preS1/preS2/S ORF
ntd 2850 - 837
POL ORF
ntd 2309 - 1625
pre C/C ORF
ntd 1816 - 2454
X ORF
ntd 1376 - 1576
gap
ntd 1427 - 1603
