# Supplementary material for: The paradox of HBV evolution as revealed from a 16th century mummy
Source: PLoS Pathog. 2018 Jan 4;14(1):e1006750. doi: 10.1371/journal.ppat.1006750 (PMC5754119; doi:10.1371/journal.ppat.1006750)
Supplement: S2 Fig — Genomic organization of overlapping open reading frames and approximate location of single-stranded portion of plus strand are indicated, as well as the relative GC (blue) to AT (green) content of the genome of X65257 and the likely location of CpG islands (light green). (PDF) [file ppat.1006750.s002.pdf]

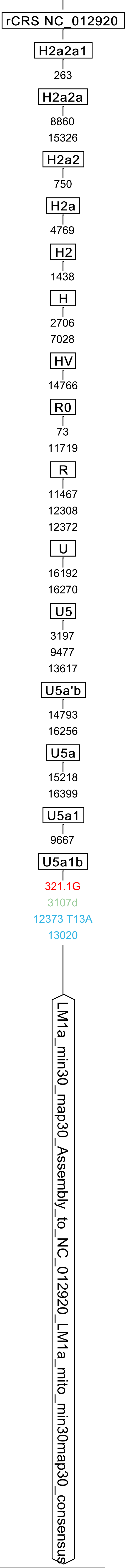

KEY
Hotspot
Local private mutation
Global private mutation
@ = assumed back mutation
or missing mutation
Heteroplasmic mutation
