# Supplementary figures and images for: Correction: The paradox of HBV evolution as revealed from a 16th century mummy
Source: PLoS Pathog. 2018 Feb 9;14(2):e1006887. doi: 10.1371/journal.ppat.1006887 (PMC5806894; doi:10.1371/journal.ppat.1006887)

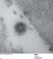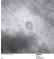

Supplement: S1 Fig — (PDF) [file ppat.1006887.s001.pdf]

HBV reads from LM1 mapped to X65257 (min30, MQ30)

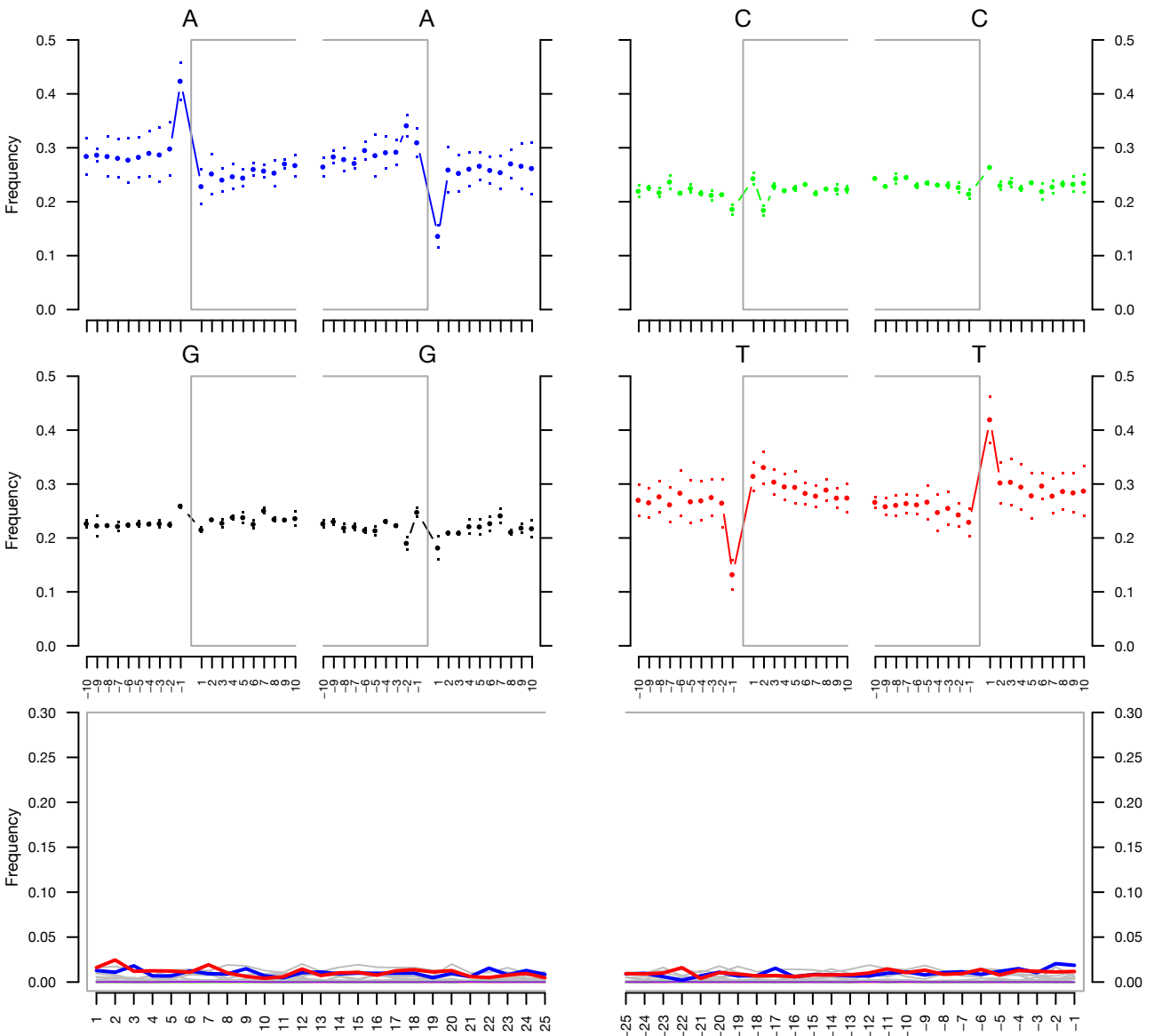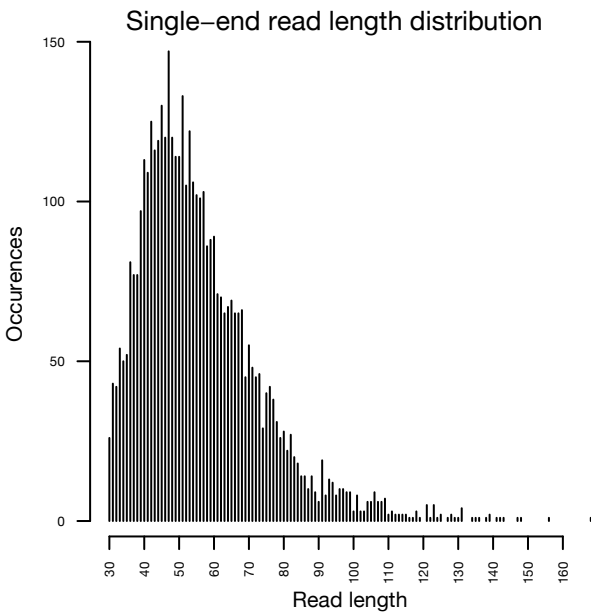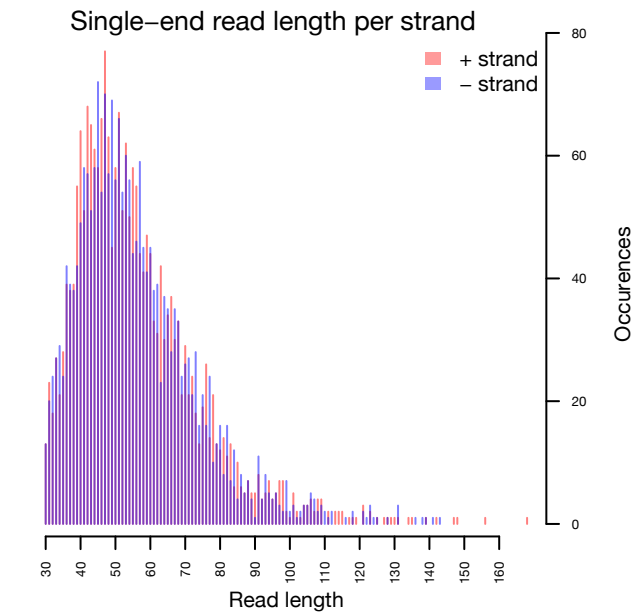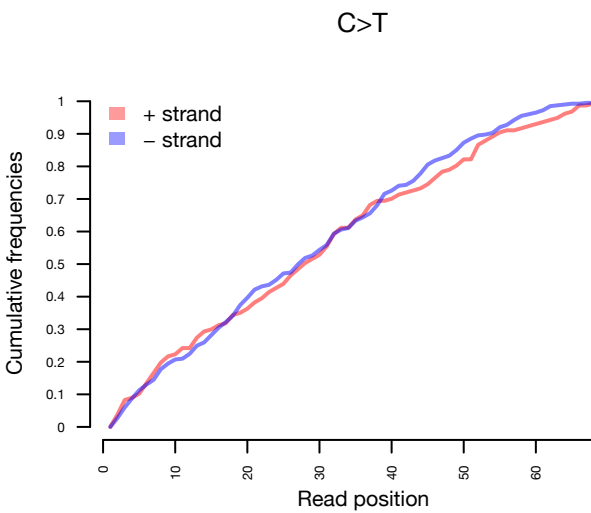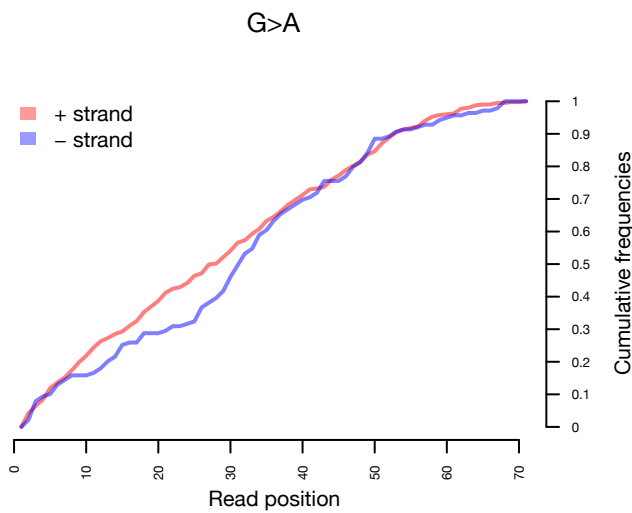

Supplement: S4 Fig — (PDF) [file ppat.1006887.s004.pdf]

**A**

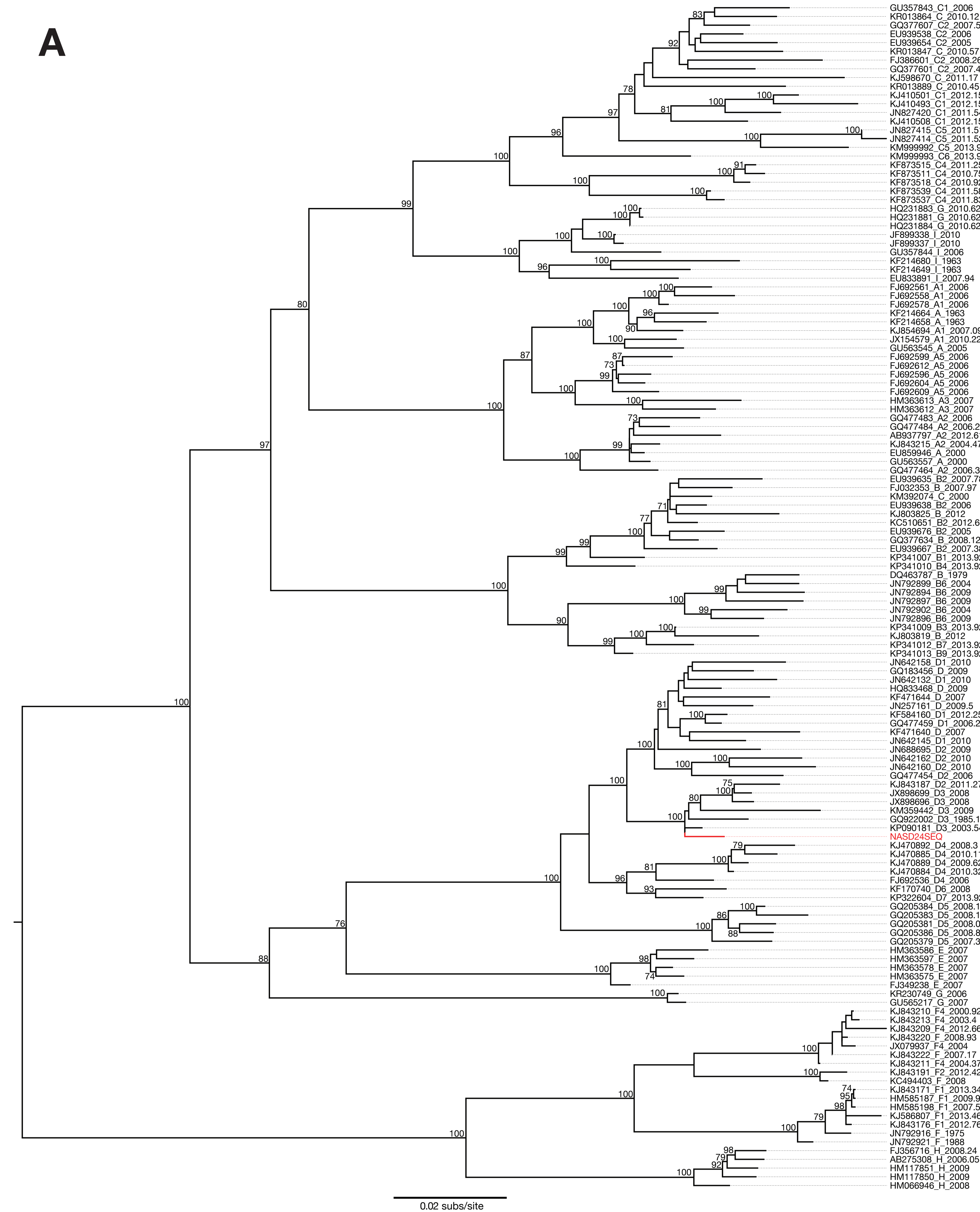

## ITALIAN

# B

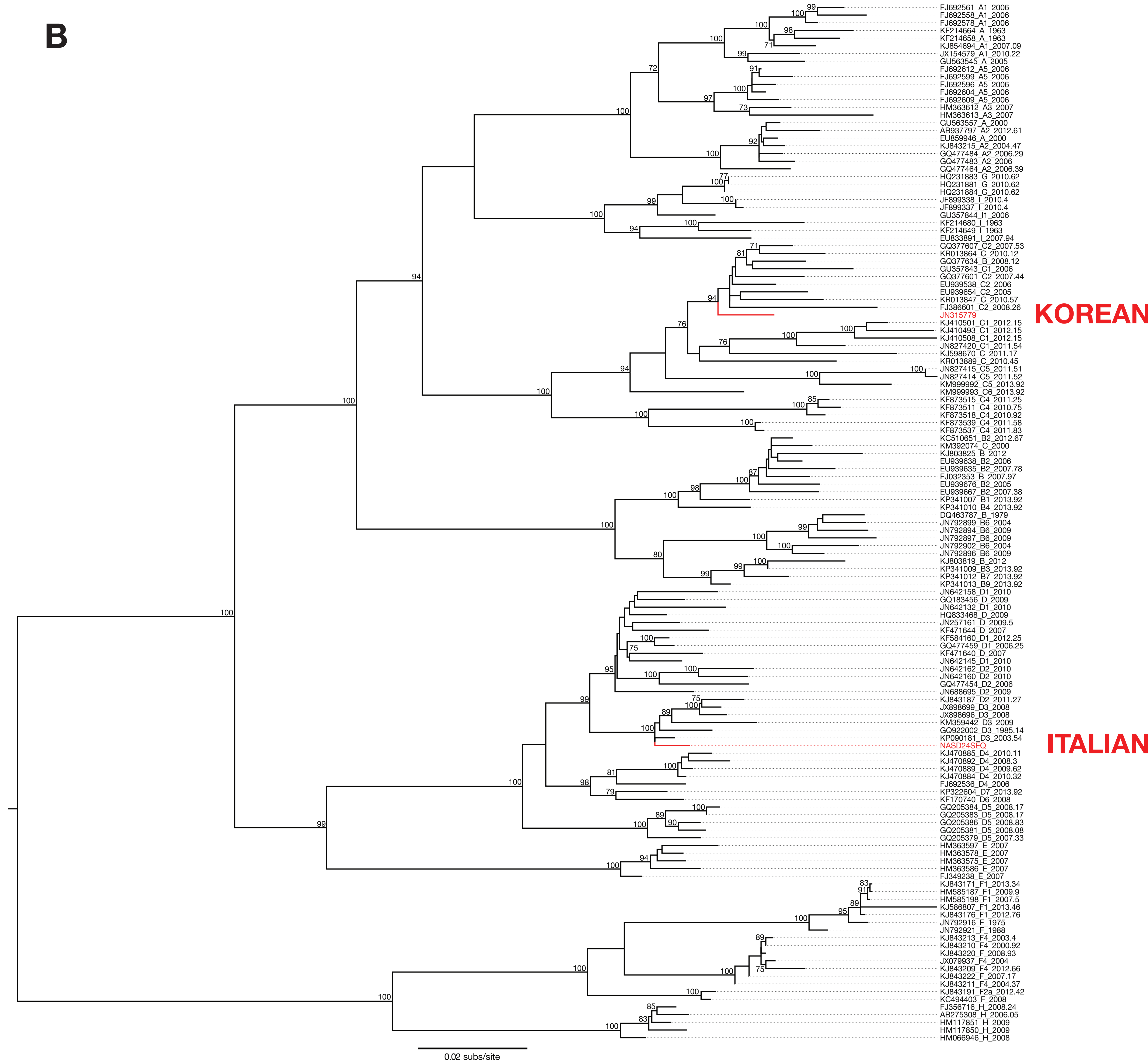

## KOREAN

**ITALIAN**

C

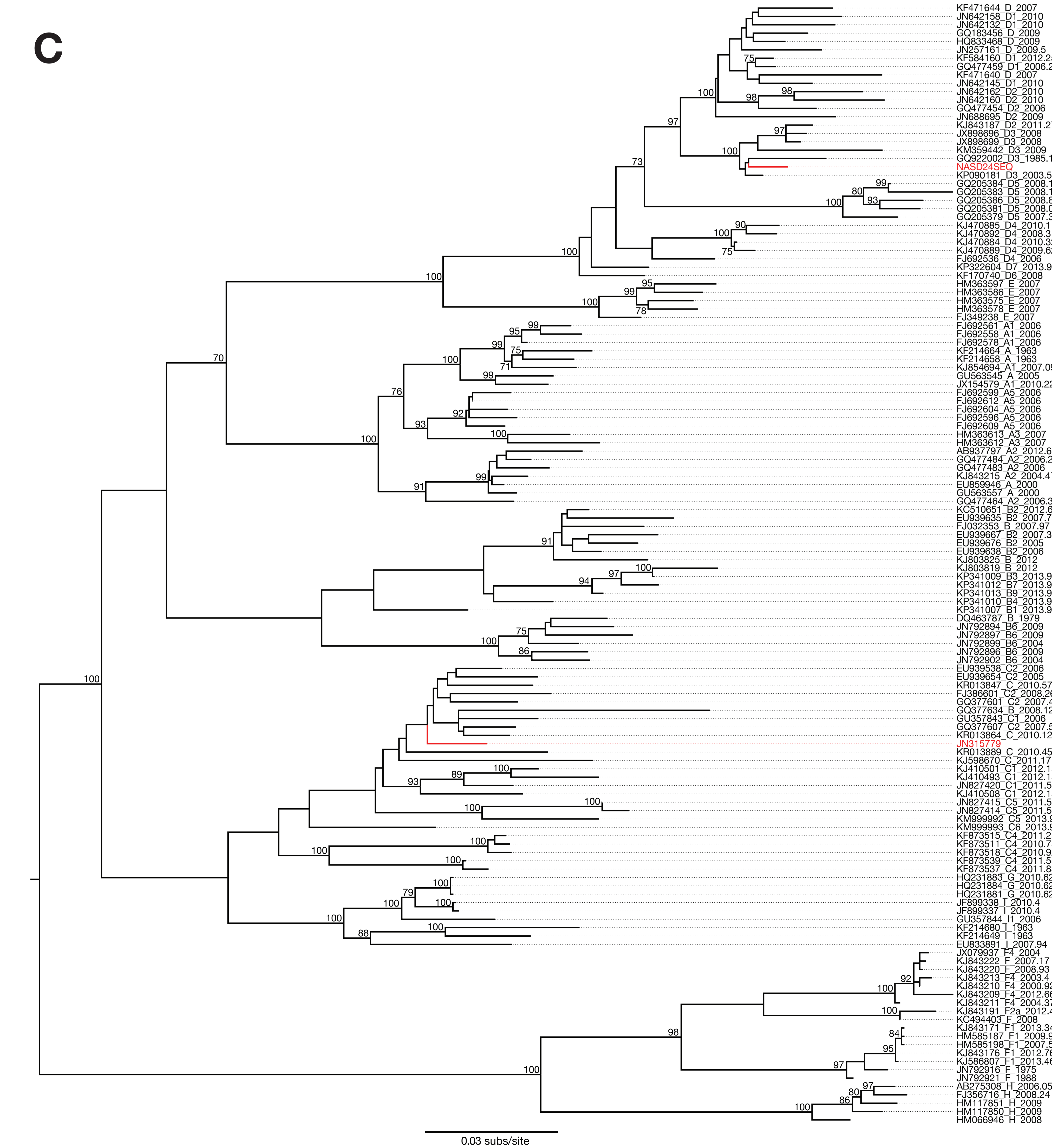

# ITALIAN

## KOREAN

D

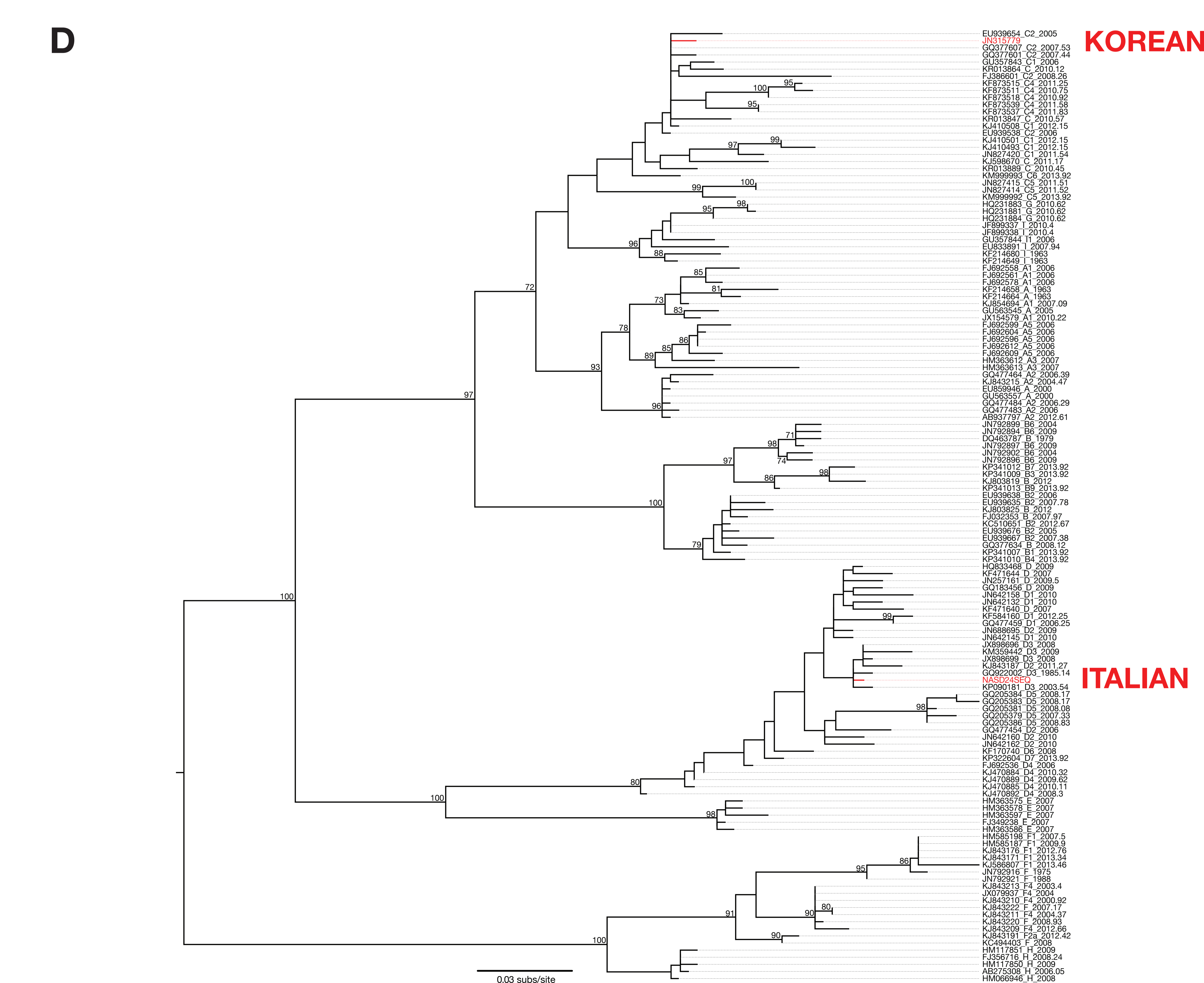

## KOREAN

ITALIAN

Supplement: S5 Fig — (PDF) [file ppat.1006887.s005.pdf]

**A**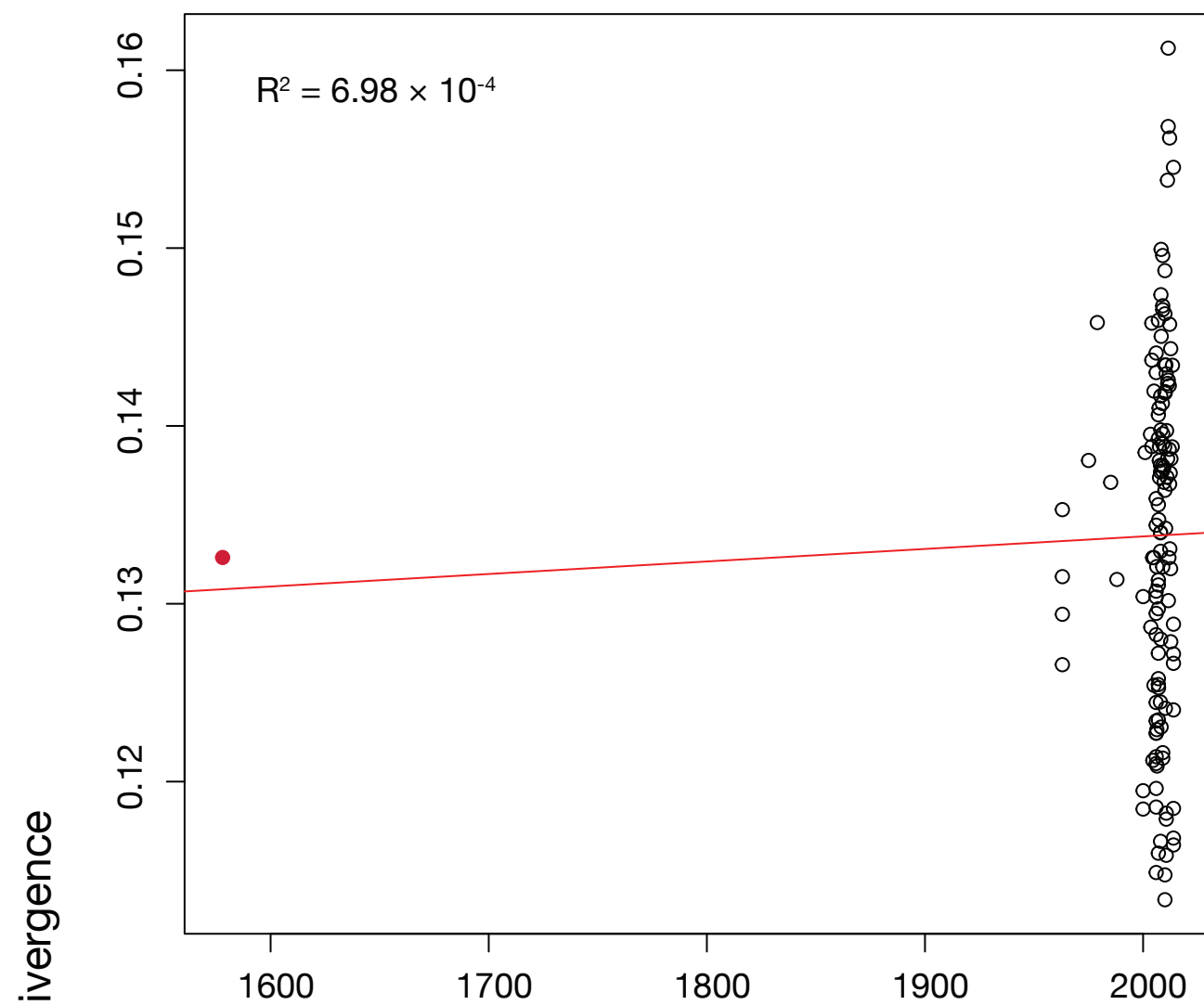**B**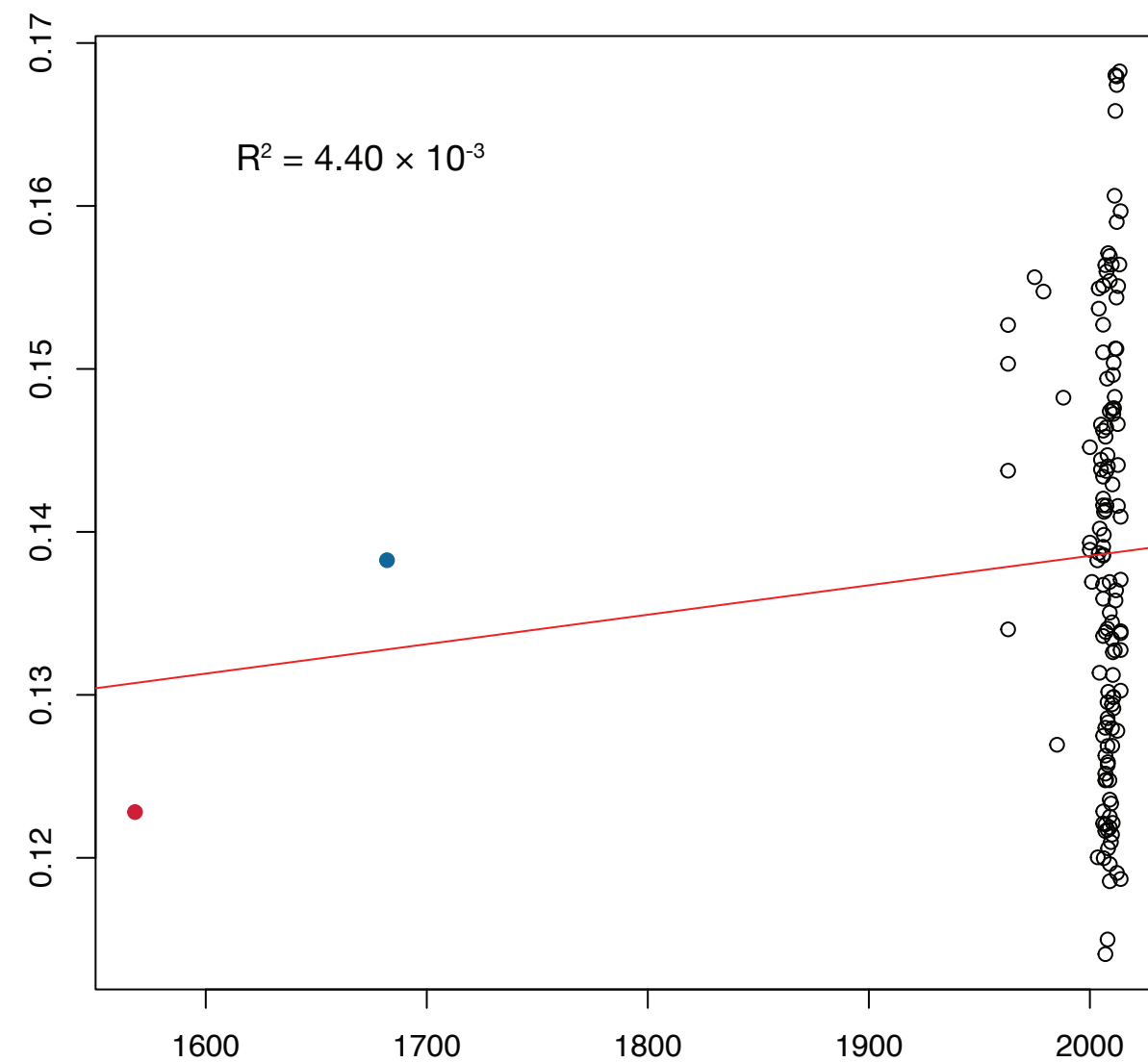**C**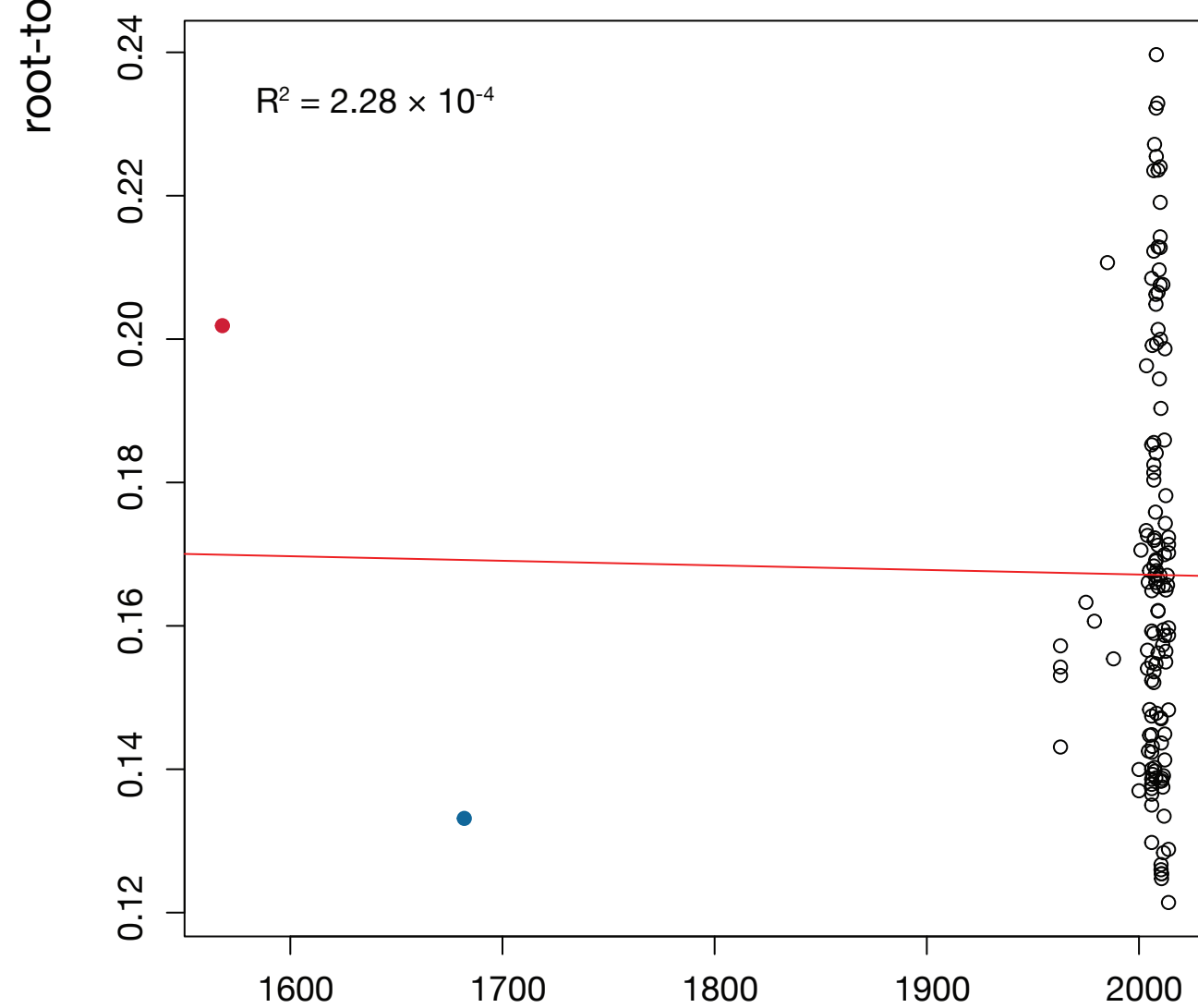**D**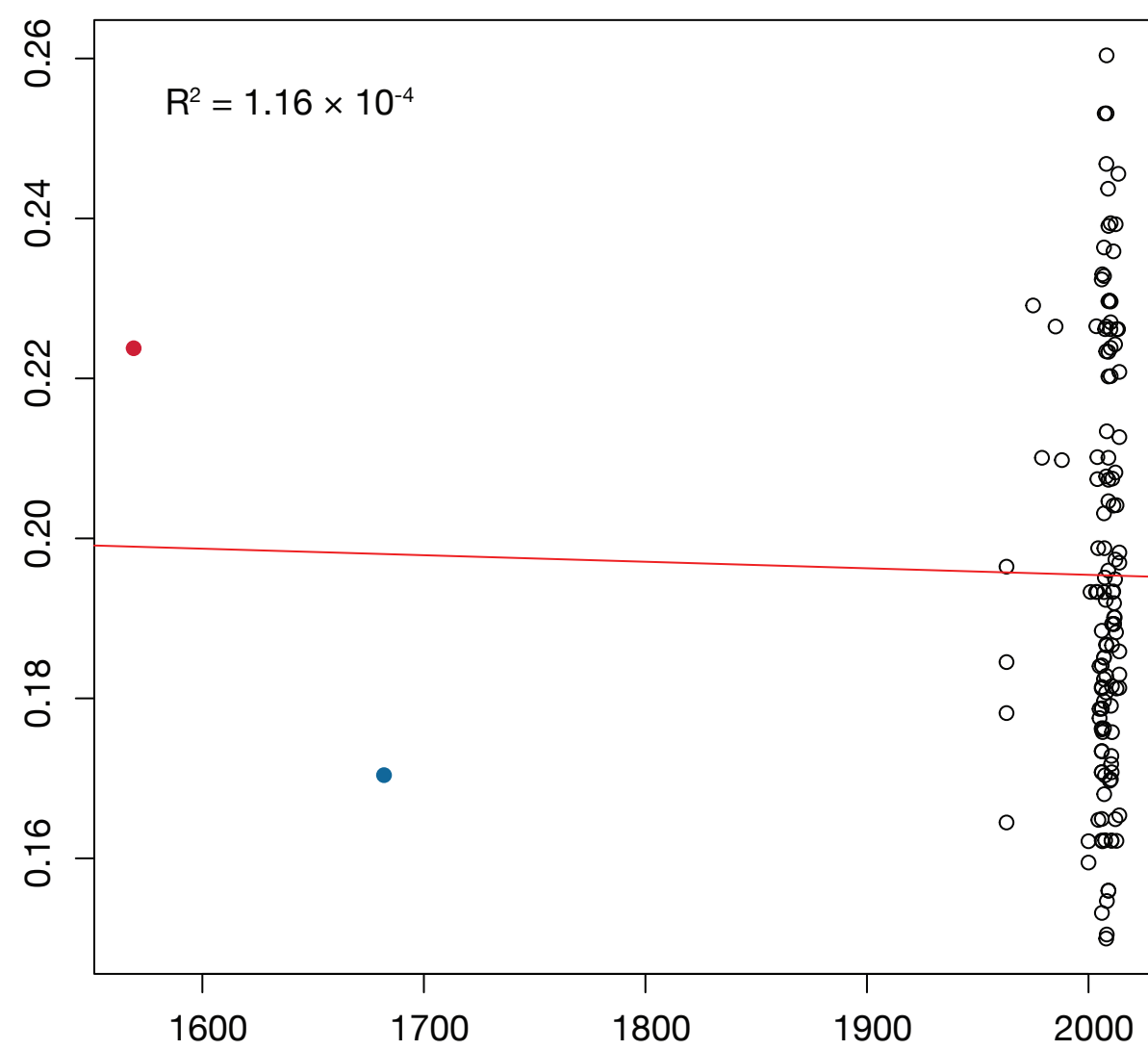

root-to-tip divergence

sampling date (year C.E.)

Supplement: S6 Fig — (PDF) [file ppat.1006887.s006.pdf]

**A**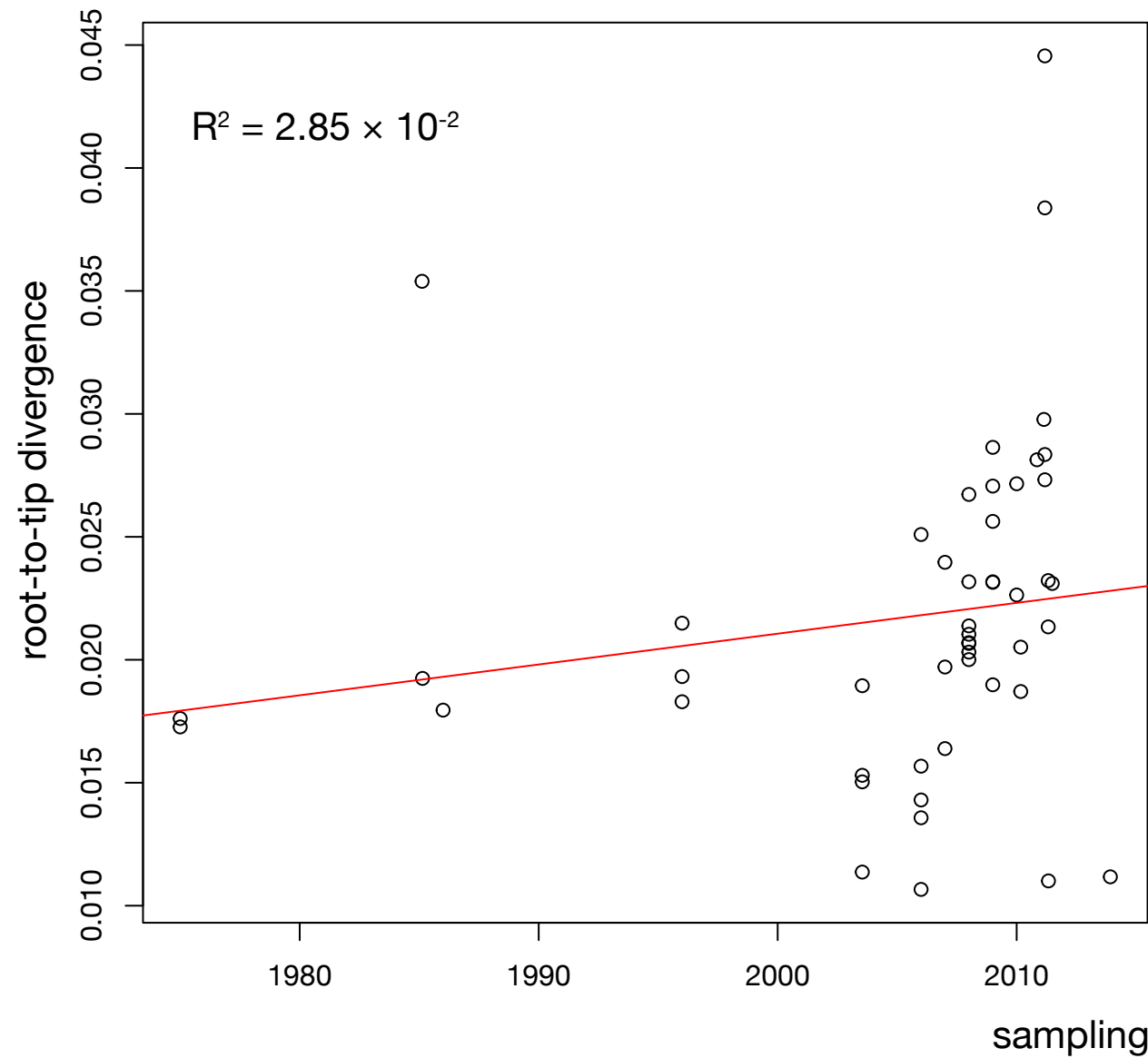**B**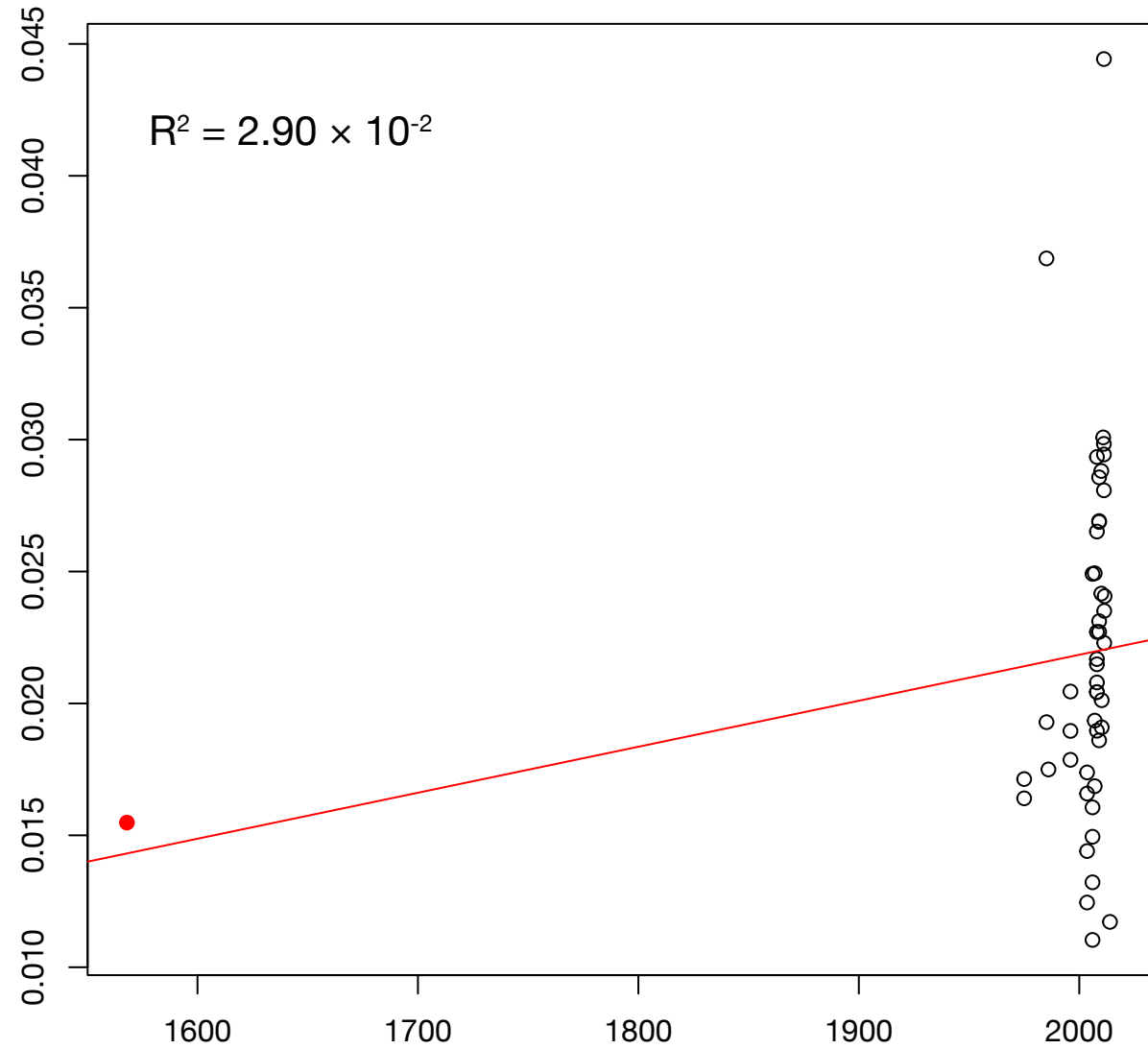

Supplement: S7 Fig — (A) Displays the D3 subgenotype. (B) Displays the D3 subgenotype with the addition of NASD24SEQ. (PDF) [file ppat.1006887.s007.pdf]

**A**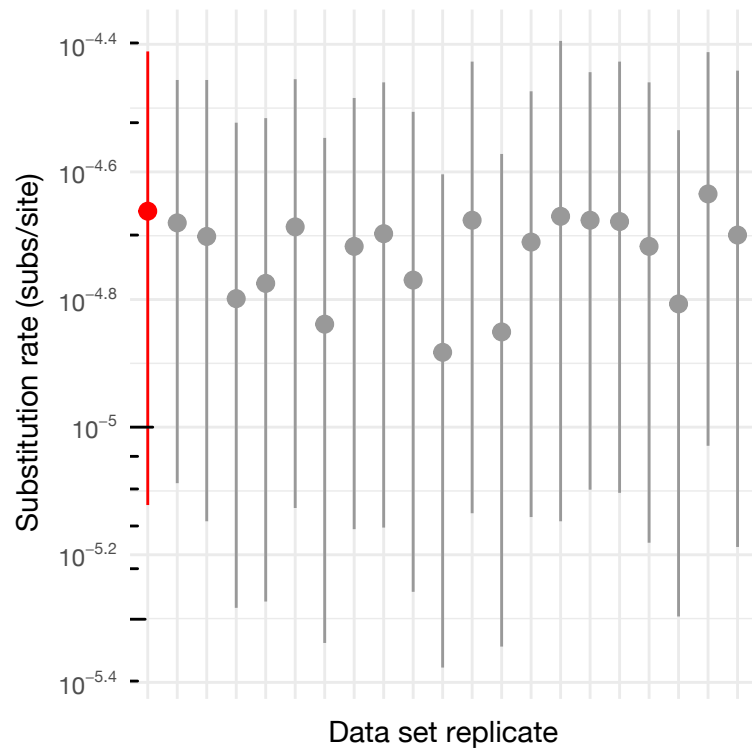**B**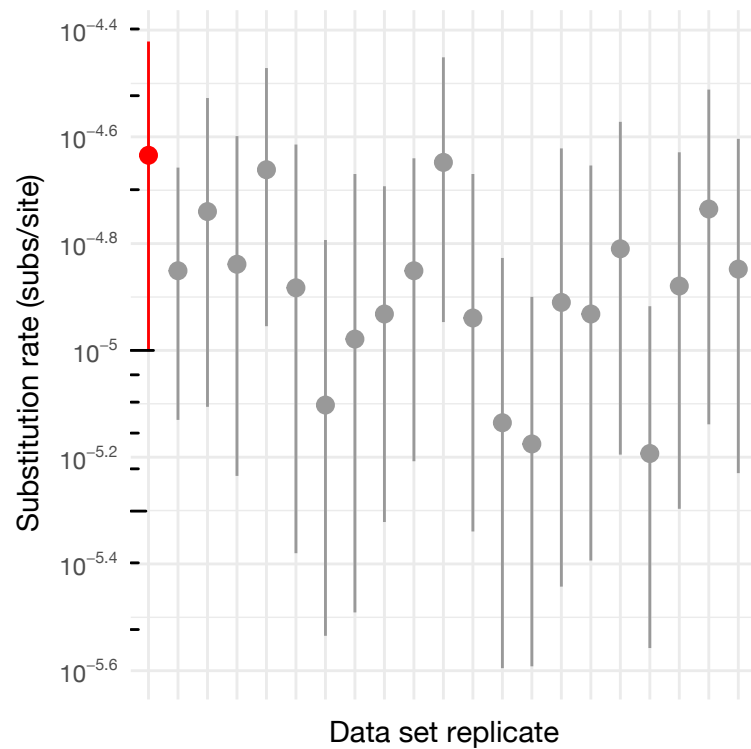**C**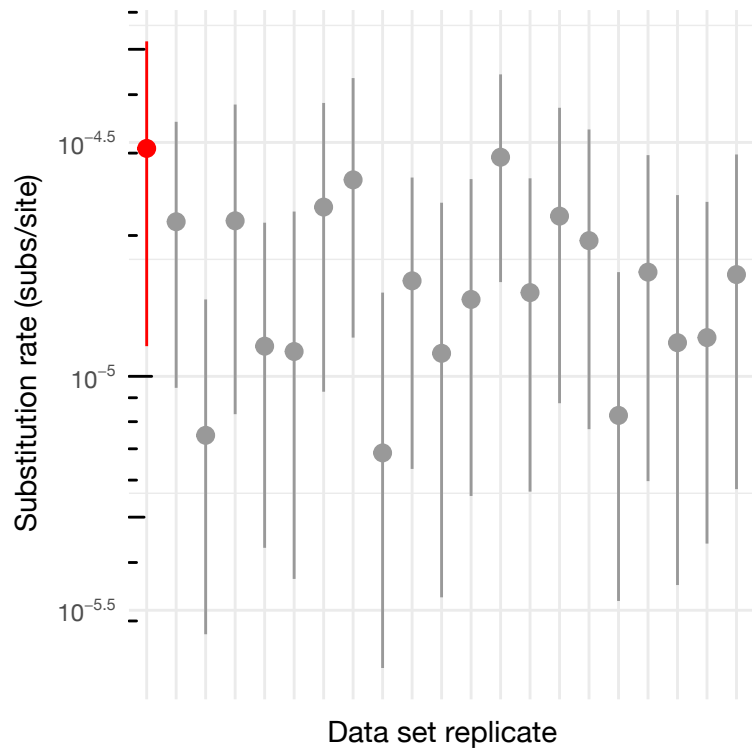**D**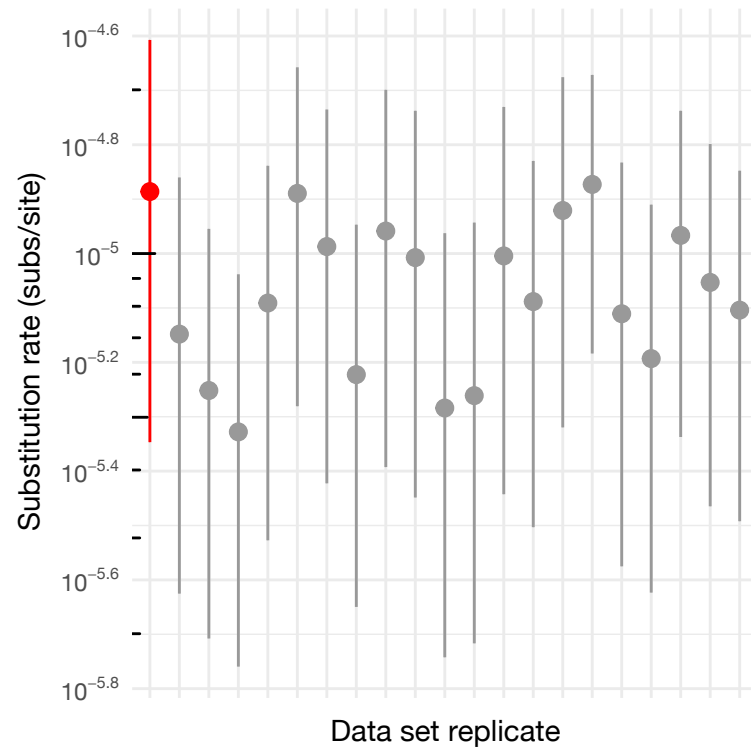

Supplement: S8 Fig — (PDF) [file ppat.1006887.s008.pdf]

**A**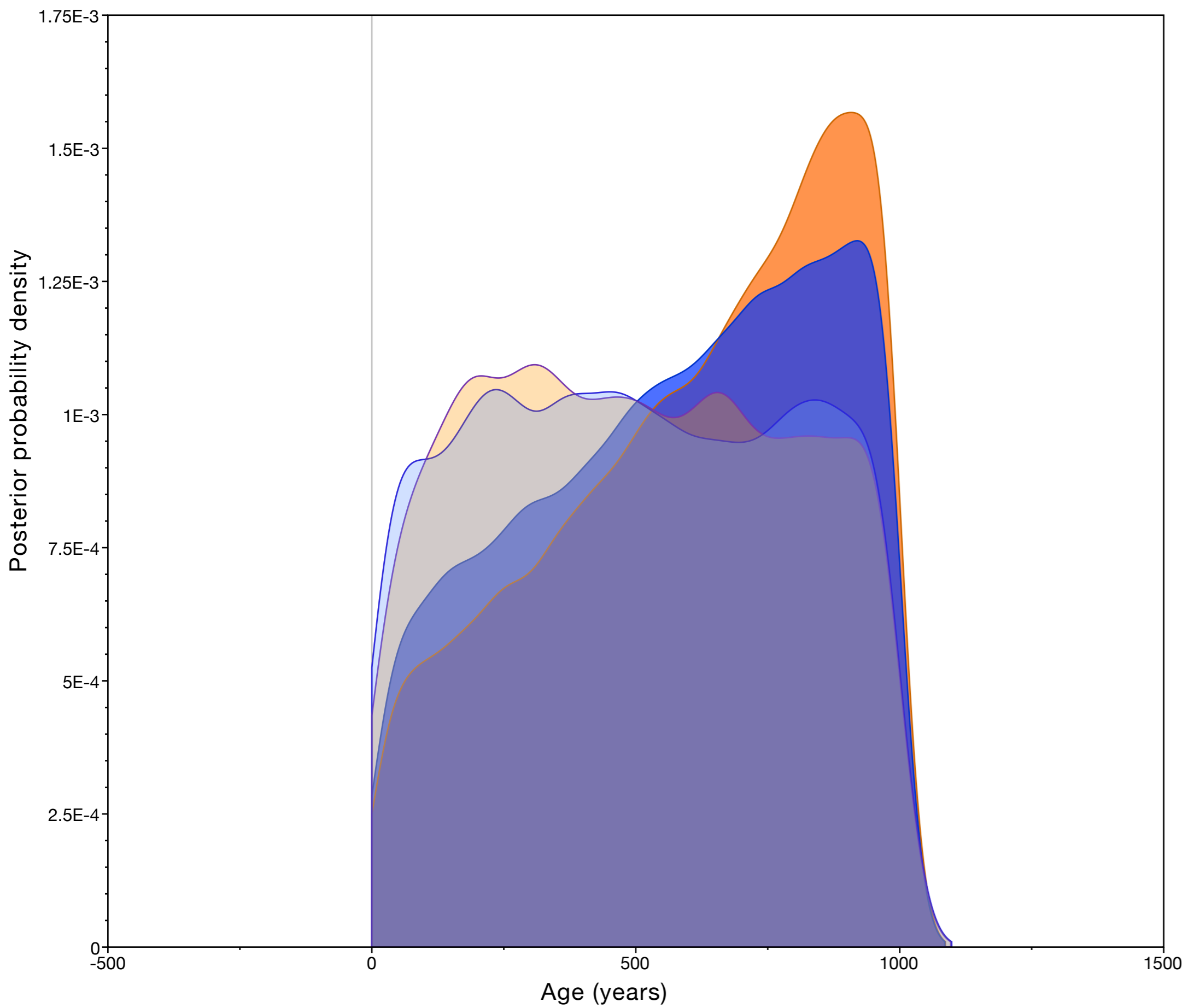**B**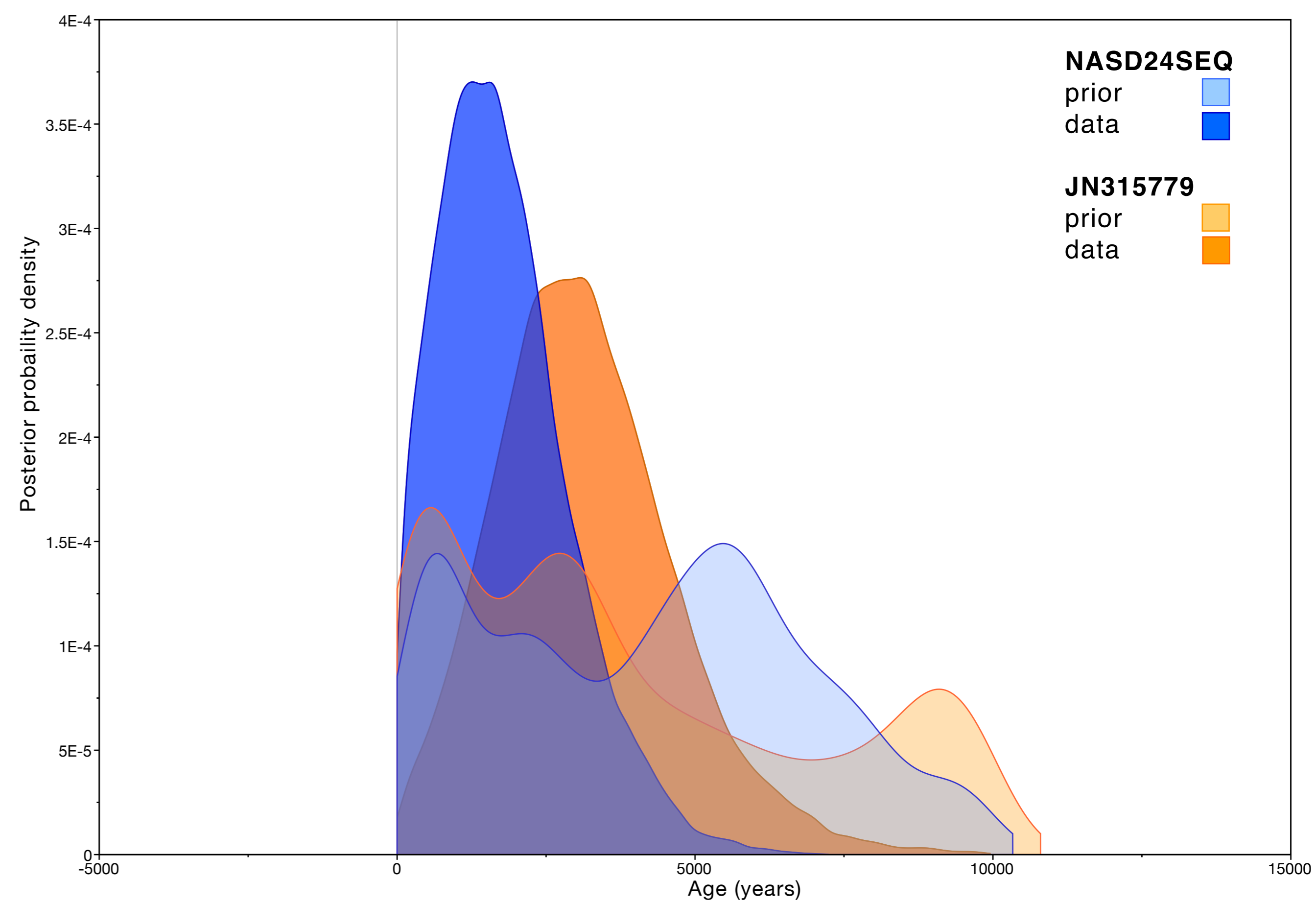

Supplement: S9 Fig — Histogram showing the probability density estimation distributions for the Bayesian analysis of NASD24SEQ and JN315779 both with and without sequence data from subset a-ii and with the internal calibration scheme using the estimation of entry into the Americas from Llamas et al. 2016 [55] to calibrate the node separating genotypes F and H. (A) With a uniform prior bounded by 0 and 1,000 years for the samples. (B) With a uniform prior bounded by 0 and 10,000 years. (PDF) [file ppat.1006887.s009.pdf]
